# Adaptive silviculture for climate change in North American forests: Operationalizing the resistance–resilience–transition framework

**DOI:** 10.1002/eap.70302

**Published:** 2026-09-20

**Authors:** Peter W. Clark, Anthony W. D'Amato, Linda Nagel, Brian J. Palik, Mike A. Battaglia, Maria K. Janowiak, Courtney L. Peterson, Christopher W. Swanston, Maria R. Vicini

**Affiliations:** ^1^ Rubenstein School of Environment and Natural Resources University of Vermont Burlington Vermont USA; ^2^ Department of Forest Resources University of Minnesota St. Paul Minnesota USA; ^3^ USDA Forest Service Northern Research Station Grand Rapids Minnesota USA; ^4^ USDA Forest Service Rocky Mountain Research Station Fort Collins Colorado USA; ^5^ USDA Forest Service Northern Research Station Houghton Michigan USA; ^6^ Forest and Rangeland Stewardship Colorado State University Fort Collins Colorado USA; ^7^ Save the Redwoods League McKinleyville California USA

**Keywords:** adaptation, assisted migration, complexity, ecological forestry, resilience, science coproduction

## Abstract

The impacts of global climate change have compelled forest managers to evaluate their practices and ecosystems, considering when and how to resist change, promote resilience, or facilitate transitions. Forest adaptation has made significant strides to move from theory to practice over the last two decades, with widespread examples of intentional, thoughtful, explicit adaptation. However, very few of these examples exist within a research framework, and their underlying theories of change are unlikely to be rigorously tested. The Adaptive Silviculture for Climate Change (ASCC) Network addresses this science gap, conceived through a process of science coproduction (i.e., translational ecology) to implement a long‐term ecological monitoring network and operational‐scale (representative of routine practice) experimental trials that test the Resistance–Resilience–Transition (RRT) adaptation framework. Fourteen sites (mean size 160 ha per site) located throughout North America applied a common, replicated study design that translates the RRT framework into treatments localized to forest types, climate‐amplified stressors, and forest management regimes. The goal of the study presented here is to examine how climate‐adaptive management is implemented across diverse forest ecosystems and evaluate alignment of specific practices (e.g., compositional diversification, structural enhancement, forest assisted migration) in advancing forest adaptation. We conducted a network‐wide meta‐analysis leveraging field measurements, forest management plans, and structured prompts to assess how site‐level ecological characteristics, global change vulnerabilities, management goals, and outcomes differ among RRT treatments compared to business‐as‐usual forest management (i.e., Common Practice). Results indicate that key adaptation pathways are achieved through modifications in forest composition (e.g., species, functional identity, and adaptive potential or assisted migration), structural characteristics (e.g., relative density, harvest complexity), and achievement of multiple, diverse desired future conditions. Additionally, differences exist among RRT treatments when examined by site‐specific conditions, principally associated with dominant overstory type (e.g., angiosperm vs. gymnosperm) or the roles of fire and drought. Despite clear network‐wide trends, important differences remain among treatments at the site level, highlighting local, operational variation in theoretical adaptation strategies. The findings of this continental experiment illustrate the flexibility of the RRT adaptation framework and reinforce the need for climate adaptation to be inherently flexible in responding to local needs and new threats.

## INTRODUCTION

The impacts of global climate change are already dramatically affecting many forested ecosystems, and the continued interaction of chronic and acute effects is expected to present ever greater challenges for natural resource managers and policymakers worldwide (Dale et al., 2001; Domke et al., 2023). The uncertainty of these impacts represents one of the greatest obstacles facing forest managers seeking to maintain the delivery of key ecosystem services while adapting to new and changing conditions. In response, conceptual and applied frameworks for climate change adaptation in forests have been proposed, with an increasing number and diversity of examples of their use in on‐the‐ground forest management activities (Janowiak et al., 2014; Millar et al., 2007; Nagel et al., 2017, 2025; Ontl et al., 2018; Rissman et al., 2018; Schuurman et al., 2022, 2025; St‐Laurent et al., 2021; Swanston et al., 2016; Verkerk et al., 2020). Although forest management and silvicultural practices—that is, the on‐the‐ground actions to manage forest stands—have been used for centuries to achieve economic, cultural, and ecological benefits, there are few examples as to how climate adaptation can be employed throughout key forest regions to address the effects of global change (D'Amato & Palik, 2021).

Adaptation has been conceptualized by various means in terrestrial ecosystems (Falk et al., 2022; Gunderson et al., 2010; Palik et al., 2020; Seidl & Turner, 2022), yet the capacity of a system to retain core functions and traits under climate change and associated perturbations may be strongly linked to local conditions. In forests, adaptive capacity can be measured in several ways (Carpenter et al., 2001; Seidl et al., 2016; Seidl & Lexer, 2013) including but not limited to tree composition and diversity (Thurman et al., 2020), forest structure and complexity (D'Amato et al., 2013; Wikle & D'Amato, 2023), variability in functional attributes and niche space occupancy (Boisvert‐Marsh et al., 2020), and/or stand heterogeneity or landscape level network connectivity (Messier et al., 2019). Concurrently, these components may be promoted or modified through deliberate silvicultural activities like harvests to alter tree density or stand structural components, modify light levels to facilitate regeneration, or tree planting to artificially promote desired species and genotypes.

In responding to local conditions, the management tactics used may vary by environmental drivers such as forest type (i.e., dominant overstory composition), climatic or edaphic factors (i.e., precipitation, soils), land use and management history, or key biotic and abiotic natural disturbance agents (i.e., insects, fire). Management actions may also focus on modifying or improving adaptive capacity in response to anticipated global change vulnerabilities and shifting conditions (e.g., pests and pathogens, drought, wildfire). Understanding the linkages between forest management, adaptive capacity, and their variability across diverse forest conditions and climate change vulnerabilities is important for determining how the forests of today will shape the forests of tomorrow and their associated functions (D'Amato et al., 2023; Seidl et al., 2016).

**FIGURE 1:**
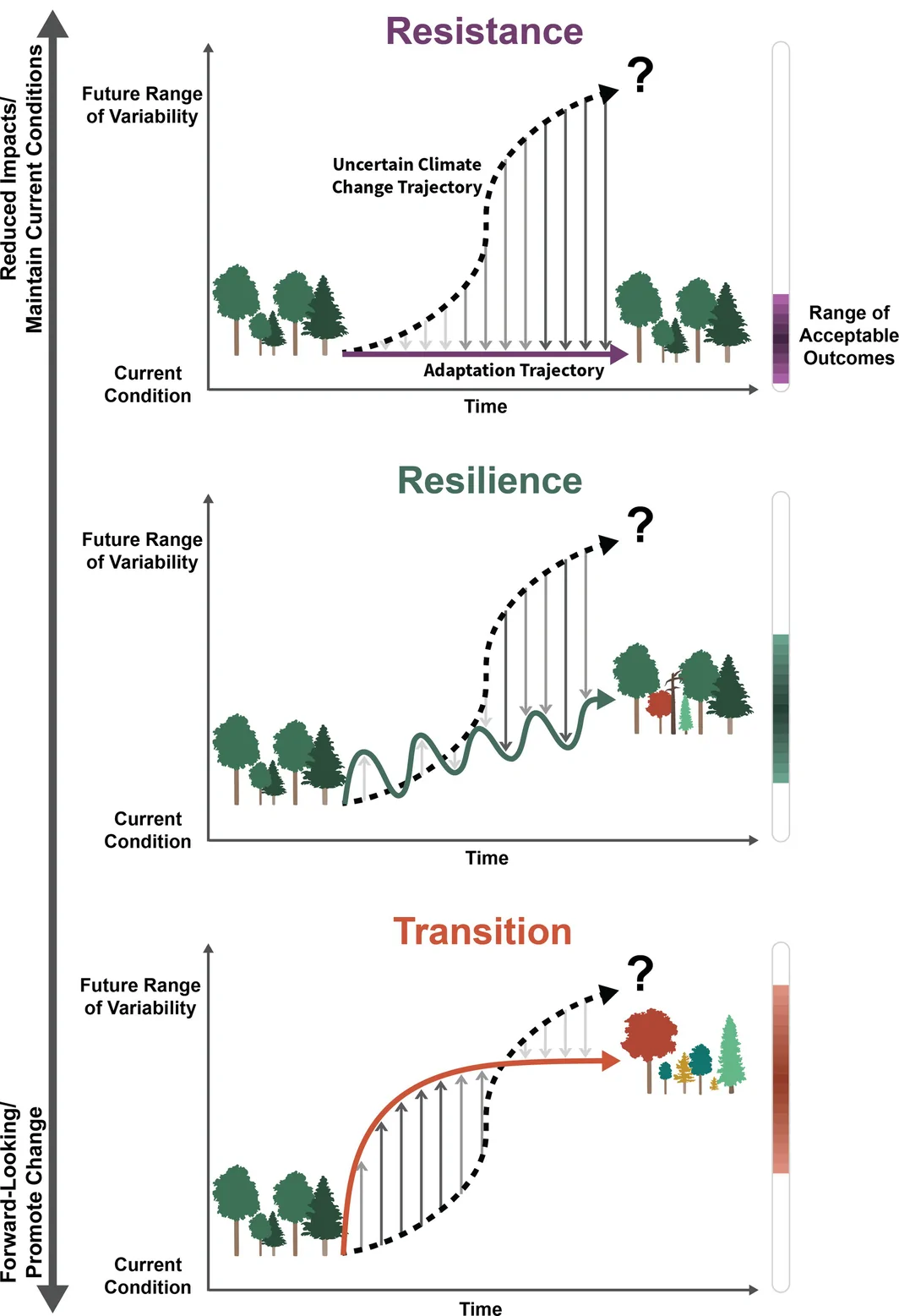
Conceptual diagram of the Resistance–Resilience–Transition (RRT) framework applied to the adaptive silviculture for climate change (ASCC) Network (see Nagel et al., 2017, 2025 for more detail), positioned vertically along a spectrum of ecological maintenance/persistence (top) to adaptation/change (bottom). Within panels, the *y*‐axes present the degree of alignment with current conditions relative to future range of variability (Seidl et al., 2016). Simplified diagrams present forest conditions in time relative to current (left) and idealized desired future conditions (right). The treatment specific adaptation trajectory is presented relative to a theorized climate change trajectory (dotted line). Vertical, shaded arrows illustrate the comparative effort (i.e., risk, cost, resources) to maintain treatments relative to climate change. A generalized distribution of the range of acceptable outcomes is presented on the right of each treatment panel. Graphic produced with support by Kailey Marcinkowski (Northern Institute of Applied Climate Science). All ASCC sites contain an unmanaged, No Action control treatment which is not explicitly examined in the work presented here. Moreover, this work does examine a business‐as‐usual active regional forest management scenario (termed: Common Practice). Both scenarios are intentionally omitted from this conceptual diagram.

Most of the various climate adaptation frameworks used in forestry stem from a shared progenitor, namely Millar et al. (2007), whose seminal work was the first to propose how an adaptation framework may be applied in forested ecosystems along a continuum of change and adaptability. They established common usage of the terms *Resistance*, *Resilience*, and *Response*, which were further refined and clarified to *Resistance*, *Resilience*, and *Transition* (RRT) by Swanston et al. (2016) and Nagel et al. (2017, 2025) to be more readily communicated to a management audience. The RRT framework broadly conceptualizes how a suite of adaptation strategies may be employed in forest management for global change (see Figure 1 for conceptual diagram). Here, Resistance is defined as actions that improve the defenses of the forest against anticipated change or directly defend the forest against disturbance to maintain relatively unchanged conditions. Resilience is defined as actions that accommodate some degree of change but encourage multiple, diverse pathways for a forest to recover after a disturbance and return to a prior or other desired reference condition after disturbance. Finally, Transition is defined as actions that intentionally facilitate change and enable ecosystems to adaptively respond to shifting or new conditions. Although presented as discrete options, these strategies more accurately exist along a gradient of change and may include related goals, tactics, and outcomes when applied among different forest types. Since the development of RRT, other adaptation frameworks have been proposed (cf. Resist‐Accept‐Direct [RAD]; Schuurman et al., 2022, 2025) or refined upon (Climate‐Smart Forestry; Verkerk et al., 2020) that are widely accepted for conservation of forested ecosystems. The concepts presented by these frameworks have been broadly adopted and implemented by management communities responding to the speed of change and urgency of response, but their very novelty requires rigorous scientific and empirical testing that yet lags far behind implementation (Janowiak et al., 2014; Ontl et al., 2018; Stein et al., 2014; St‐Laurent et al., 2021; Swanston et al., 2016; U.S.G.S., 2025). Moreover, outside of local evaluations, there is a need to understand how adaptation approaches differ and/or converge when examined across a diversity of forested ecosystems.

**FIGURE 2:**
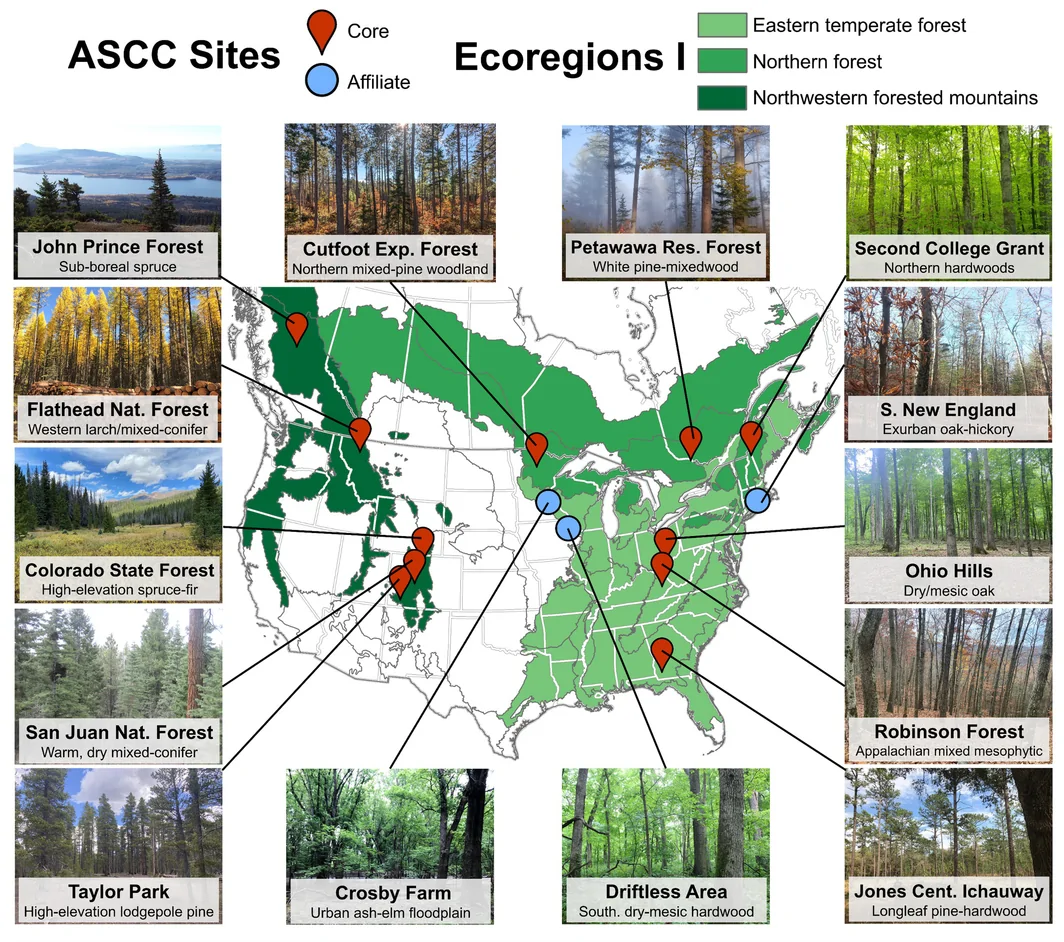
The distribution of the adaptive silviculture for climate change (ASCC) core and affiliate sites in the United States and Canada, relative to North America ecoregions. Type I ecoregions represented in the study are highlighted. Outlines of type II ecoregions are also shown in gray. See Commission for Environmental Cooperation (1997) for comprehensive list of ecoregions. Photographs courtesy of the ASCC Network (https://adaptivesilviculture.org/).

Large‐scale long‐term ecological monitoring networks and coordinated experiments have proven essential in addressing basic and applied issues in forested ecosystems across space and time (Hughes et al., 2017; Lindenmayer et al., 2012). Within these networks, a common experimental design, standardized protocols, and/or research questions are pursued across a wide ecologic and geographic network yet are localized to ecosystems or regional management regimes. The Adaptive Silviculture for Climate Change (ASCC) experiment was conceived in 2009 based on a common need identified by natural resource managers and forest scientists for on‐the‐ground, operational‐scale experiments (i.e., representative of routine forest management scale and practice) to demonstrate and empirically evaluate climate adaptation theory in practice (Nagel et al., 2017, 2025). The ASCC project is a collaborative effort among natural resource managers, scientists, and other partners and stakeholders to coproduce and establish a series of replicated experimental silvicultural trials that utilize a common experimental design but localized to diverse forest ecosystem types throughout the United States and Canada (Figure 2). Science coproduction (also referred to as translational ecology) is a collaborative process in which researchers, practitioners, and community members jointly develop knowledge, ensuring that scientific outputs are both useful and usable for real‐world decision‐making (Enquist et al., 2017). All ASCC sites use this approach to test replicated active forest management treatments based on the RRT adaptation framework (plus a passive, unmanaged *No Action* control treatment, which is not examined here; see Nagel et al. (2025) Table 1 for more information). Approximately 15 years into its development, the ASCC experiment serves as an important benchmark for testing adaptation theory and translating practices into on‐the‐ground management.

**TABLE 1:** Key characteristics of adaptive silviculture for climate change study sites based on their initial (starting) conditions.

Site and location	Forest‐type group (FIA)	Dominant overstory type and species (%)	Historical role of fire	Relative density	Mean annual precip. (mm)
**Cutfoot Experimental Forest (*CEF*)**, Minnesota, USA	White–Red–Jack Pine	**Gymnosperm** *Pinus resinosa* (85), *Pinus strobus* (6)	Extremely influential (4)	77%	675.1
**Crosby Farm Regional Park (*CFP*)**, Minnesota, USA	Elm–Ash–Cottonwood	**Angiosperm** *Populus deltoides* (50), *Ulmus americana* (10), *Acer saccharinum* (10)	Slightly influential (1)	22%	845.6
**Colorado State Forest (*CSF*)**, Colorado, USA	Spruce–Fir	**Gymnosperm** *Abies lasiocarpa* (72), *Picea engelmannii* (21), *Pinus contorta* (7)	Very influential (3)	52%	617.2
**Driftless Area (*DRI*)**, Iowa, Minnesota, and Wisconsin, USA	Oak–Hickory	**Angiosperm** *Quercus rubra* (40), *Quercus alba* (20), *Acer saccharum* (8)	Extremely influential (4)	70%	985.5
**Flathead National/ Coram Experimental Forest (*FNF*)**, Montana, USA	Western Larch	**Gymnosperm** *Larix occidentalis* (72), *Abies lasiocarpa* (7), *Pseudotsuga menziesii* (7)	Extremely influential (4)	43%	425.7
**Jones Center at Ichauway (*JCI*)**, Georgia, USA	Longleaf–Slash Pine	**Gymnosperm** *Pinus palustris* (85), *Quercus margarettae* (3.5), *Quercus falcata* (2.8)	Extremely influential (4)	23%	1334.8
**John Prince Research Forest (*JPF*)**, British Columbia, CA	Spruce–Fir	**Gymnosperm** *Picea glauca* (33), *Abies lasiocarpa* (25), *Pseudotsuga menziesii* (17)	Very influential (3)	52%	554.0
**Ohio Hills (*OHH*)**, Ohio, USA	Oak–Hickory	**Angiosperm** *Quercus alba* (34.1), *Quercus montana* (12.6), *Quercus velutina* (11.8)	Extremely influential (4)	76%	1150.4
**Petawawa Research Forest (*PRF*)**, Ontario, CA	White–Red–Jack Pine	**Gymnosperm** *Pinus strobus* (65), *Pinus resinosa* (20), *Populus tremuloides* (10)	Very influential (3)	33%	985.0
**Robinson School Forest (*RSF*)**, Kentucky, USA	Oak–Hickory	**Angiosperm** *Acer rubrum* (23), *Quercus alba* (16), *Fagus grandifolia* (15)	Very influential (3)	89%	1294.1
**Second College Grant (*SCG*)**, New Hampshire, USA	Maple–Beech–Birch	**Angiosperm** *Acer saccharum* (64.3), *Fagus grandifolia* (14.6), *Betula alleghaniensis* (11)	Not at all influential (0)	63%	1132.8
**San Juan National Forest (*SJC*)**, Colorado, USA	Ponderosa Pine	**Gymnosperm** *Abies concolor* (37), *Pinus ponderosa* (31), *Pseudotsuga menziesii* (26)	Extremely influential (4)	45%	287.0
**Southern New England Exurban (*SNE*)**, Connecticut and Rhode Island, USA	Oak–Hickory	**Angiosperm** *Quercus velutina* (19), *Acer rubrum* (17), *Quercus alba* (12)	Slightly influential (1)	66%	1195.1
**Taylor Park (*TPC*)**, Colorado, USA	Lodgepole Pine	**Gymnosperm** *Pinus contorta* (93), *Picea engelmannii* (6), *Abies lasiocarpa* (1)	Extremely influential (4)	40%	477.5

The aim of this study is to use the ASCC Network to examine how climate adaptation concepts are translated into on‐the‐ground active forest management across diverse forest ecosystems in North America, by characterizing cross‐site trends in treatments and evaluating how local forest types, vulnerabilities, and management contexts shape implementation. We primarily focus on evaluating patterns at the network scale but also examine how site‐level treatments are locally tailored to distinct forest types and vulnerabilities, reflecting ASCC's core principle that adaptation strategies must be grounded in local ecological and social contexts (Nagel et al., 2025). We achieve this by comparing how experimental RRT treatments and a “business‐as‐usual” regional forest management scenario (hereafter, Common Practice) relate to current site conditions, vulnerabilities, and management objectives and/or desired future ecological conditions. Our specific research questions are:

1. How do site conditions and global change vulnerabilities differ across the ASCC Network?
2. How do site conditions and vulnerabilities influence how RRT treatments are broadly applied (relative to Common Practice) to meet locally relevant management goals, tactics, and desired future conditions?
3. How do RRT treatments differ or converge at the Network‐level as well as when localized among different ecosystem types (and associated vulnerabilities)?
4. What roles do climate adaptation silvicultural practices like forest assisted migration play in sites and in the application of RRT treatments?

## METHODS

### ASCC experimental design

The ASCC Network currently consists of 14 installations located on public and private lands in rural, urban, and exurban settings and includes National Forests, state forests, government‐led (United States and Canada) Experimental Research Forests, university research forests, private forests, and a regional urban park. Presently, the ASCC Network collectively covers over 2000 ha of forest lands in the United States and Canada (Table 1). Installations are located within six North American Type II Ecoregions (Figure 2; Commission for Environmental Cooperation, 1997) and among nine dominant forest‐type groups, based on USDA Forest Service Forest Inventory and Analysis cover types (Gray et al., 2012). Although key differences exist within groups sometimes consisting of several forest types, the nine generalized forest‐type groups represented here include Oak–Hickory (4 sites, forest‐type covers 570,964.9 km^2^ in the continental United States alone), Spruce–Fir (2 sites, 64,775.4 km^2^), White–Red–Jack Pine (2 sites, 41,280.5 km^2^), Elm–Ash – Cottonwood (1 site, 95,741.8 km^2^), Lodgepole Pine (1 site, 56,831.7 km^2^), Longleaf–Slash Pine (1 site, 51,001.9 km^2^), Maple–Beech–Birch (1 site, 190,721.9 km^2^), Ponderosa Pine (1 site, 92,817.0 km^2^), and Western Larch (1 site, 7466.4 km^2^; USDA, 2024).

A key feature of the ASCC Network approach is to maintain and apply a common experimental design and RRT treatment framework consistently across all installations. Despite these commonalities, treatments were tailored to local site conditions through science coproduction that integrates local ecology, regional forest management practices, and climate adaptation strategies in consideration of predicted future vulnerabilities linked to global change. Each of the 14 installations participated in a site‐specific multi‐day workshop and co‐development process to identify desired future conditions of forests to aid in treatment design, with each site utilizing the same experimental approach and study design structure for treatment types and spatial/temporal factors. Desired future conditions are defined here as the desired outcome from management that describes the expected composition, structure, and other attributes of forests and associated vegetation in the future to achieve and maintain values and identified ecological processes, which may vary from treatment to treatment (Golladay et al., 2016).

ASCC sites and treatments were designed and coproduced by individuals that, on average, represented federal agencies (33.5% SE ± 6.2), state agencies (23.2% ±5.4), academic institutions (23.1% ±3.1), nonprofit or conservation organizations (10.9% ±5.4), forest industry (6.6% ±2.5), Tribal and First Nations (1.7% ±1.1), and other entities (1.1% ±1.0). Furthermore, most sites were designed and monitored by individuals with expertise and training in forest ecology (100.0%), silviculture and forest management (100.0%), wildlife (85.7%), timber production (71.4%), wildfire management and science (64.3%), climate science (57.1%), modeling (landscape simulation, growth, and production) (57.1%) and soil science (57.1%), with lesser, localized representation in science communication (50.0%), entomology (42.9%), remote sensing and aerial imagery (42.9%), genetics (35.7%), plant physiology (35.7%), hydrology (21.4%), cultural heritage or archaeological resources (14.3%), economics (14.3%), social science and human/cultural dimensions (14.3%), fisheries (7.1%), and biogeochemistry (7.1%). See Nagel et al. (2017, 2025), and https://www.adaptivesilviculture.org/ for a comprehensive review of the intent for ASCC and the experimental framework.

ASCC Network sites and treatments subscribe to a set of minimum standards, with a few exceptions in terms of land area. Eleven “core” study sites consist of a minimum of four replicates for each treatment (see Experimental Treatment descriptions, below), with each treatment unit representing 8–10 ha in size, across a reasonably uniform forest study area (≥160 ha). Additionally, three sites (known as “affiliate” sites) are located in more fragmented and heavily human populated landscapes; these have smaller and more geographically dispersed treatment units but otherwise follow the same experimental protocols. Each site follows a common measurement protocol to assess key response variables and includes pre‐ and post‐treatment measurements, allowing for future cross‐site comparisons. The common set of core monitoring variables includes forest composition, structure, assessments of forest health, and measures of tree productivity. Moreover, many sites include additional monitoring of forest microclimatic conditions, animal, insect, and fungal communities, and ecosystem processes, like carbon storage and sequestration.

Each ASCC installation was conceived and treated opportunistically based on available resources. Therefore, sites may represent different stages of development and implementation. Despite different degrees of site‐level implementation, the data collected for our analysis were obtained in a way to capture conditions that are representative and comparable. For more information about the data collected, see *Mixed‐Methods Meta‐Analysis* below.

### Experimental treatments

Each ASCC site applied three replicated experimental treatments based on the RRT adaptation framework (Figure 1; Appendix S1: Table S1; see Nagel et al. (2025) for additional examples and treatment details). These treatments exist along a gradient of adaptation that ranges from conservative approaches that promote current ecosystem persistence to those that encourage fundamental changes in ecosystems. These conceptual treatments were then developed into silvicultural practices based on site‐specific ecosystems, informed by historical conditions and climate change vulnerabilities.

Each ASCC site also includes an unmanaged, No Action control treatment; however, these are intentionally not analyzed here given the focus of this work is on active forest management strategies. Rather, for this investigation, an alternative treatment termed Common Practice was developed to serve as a baseline scenario based on “business‐as‐usual” active forest management approaches that typify practices most commonly used in forest types in the region for each site. Common Practice treatments were based on silvicultural prescriptions outlined in regional forest silvicultural guides (i.e., Alexander, 1986; British Columbia Ministry of Forests, 2003; Gilmore & Palik, 2006; Leak et al., 2014) and refined based on expert forest manager recommendations at each site. A silvicultural prescription is a forest harvest management plan that outlines specific target levels of tree stocking (i.e., basal area) and age and size structures, species composition, and other forest demographic properties relative to baseline conditions. Therefore, this work compares four active forest management scenarios: (1) Resistance, (2) Resilience, (3) Transition, and (4) Common Practice.

Among treatments, site managers elected to employ tactics such as forest assisted migration (FAM) to support treatment outcomes (Aubin et al., 2011). Forest assisted migration is a strategy employed in response to natural plant migration rates lagging behind the rate of climate change induced shifts in species ranges, resulting in a climate mismatch over time (Sittaro et al., 2017). FAM refers to the planting of seeds and seedlings of future‐climate‐adapted species and genotypes of trees projected to increase in habitat suitability, often to compensate for a projected loss of habitat and decline of native species of commercial, cultural, and ecological values (Pedlar et al., 2012; Williams & Dumroese, 2013). Within the umbrella of FAM, different terms are commonly used to differentiate a spectrum of options, put forth by Pedlar et al. (2012). For instance, assisted population migration (aka assisted population expansion) refers to the movement of genotypes within the current (or historical) distribution of a species towards areas and habitats that are forecasted to be better adapted under changing climatic conditions (often poleward or upslope). Next, assisted range expansion commonly refers to the movement of species relatively short distances beyond historical ranges (often poleward or upslope), but to areas where the species are expected to naturally migrate into over long periods of time if migration rates kept pace. Lastly, assisted species migration is intentional long‐distance migration (e.g., interregional, transcontinental, and intercontinental) of a species beyond areas ever reasonably accessible via natural dispersal. In this manuscript, we rely on these terms to assess how FAM is applied among different ecosystems and treatment types.

### Mixed‐methods meta‐analysis

To gather insights into emergent trends in the ASCC Network, we conducted a meta‐analysis among sites which generated a standardized dataset of (1) forest and ecological conditions (e.g., species composition, stocking), (2) historical drivers and future climate vulnerabilities, and (3) the management goals and tactics used to achieve desired future conditions among treatments. We employed a mixed‐methods approach (Kemp et al., 2015; Talal & Santelmann, 2020) to compile both quantitative and qualitative data to compare differences among sites and four active forest management options: the three RRT treatments plus a Common Practice scenario for local comparison. The data used were collected by numerous site‐level collaborators and synthesized by 1–3 site leads for each installation. To collect data, a Qualtrics survey instrument was administered in the Fall of 2023 to aggregate quantitative measures and solicit qualitative responses to structured prompts (see Appendix S1: Section S1 for example survey instrument).

Quantitative data obtained for each site included measures related to forest composition (tree species and relative abundance, expressed in terms of percent basal area) and species‐level future climate adaptability, informed by species‐distribution models (e.g., Peters et al., 2020). Additionally, we obtained values related to forest stocking and structural complexity, measured in terms of basal area (m^2^/ha) and trees per hectare (>10 cm diameter at breast height), as well as residual stand conditions generated by harvest treatment patterns (e.g., gaps, patches, thinned areas, unharvested reserves) and extent of these conditions (percent of treatment unit; Puettmann et al., 2009). Pretreatment values were generated via raw field measurements. Like the Common Practice scenario, post‐treatment data were generated from target values (expressed in terms of stocking and composition) outlined from silvicultural prescriptions developed for each treatment and compared against raw field measurements. Where post‐treatment field data were lacking, we used the prescribed target values obtained from the silvicultural plans as proxies for intended stand conditions, interpreting them as explicit management objectives rather than observed outcomes.

Qualitative data were generated based on the insights of up to three site leads arranged around three themes aimed at capturing factors that (1) historically shaped site‐level ecosystem dynamics (10 prompts), (2) site‐level vulnerabilities linked to global change (18 prompts), and (3) treatment‐level management objectives and/or desired future conditions (19 prompts; see Appendix S1: Table S2 for complete list of environmental conditions, global change vulnerabilities, and management objectives). For each of the 47 prompts, ASCC site leads and coordinators ranked each factor using a 0–4 ordinal scoring system and identified factors of least to most importance or influence, where 0 = “not at all important” and 4 = “extremely important” to site or treatment outcomes. Quantitative and qualitative datasets were then merged to be able to assess links between ecological conditions, vulnerabilities, and management objectives relative to treatment outcomes to confer adaptive capacity.

### Analysis

To compare outcomes across ecologically distinct and potentially divergent sites, standardization was applied to raw forest stocking, compositional diversity, and management objective data. To examine forest structure, we calculated relative density index (RD) based on Reineke's stand density index (SDI; Reineke, 1933; Woodall et al., 2005). Relative density is a biologically relevant measure that relativizes stocking levels to the site allowing for practical comparisons across forests with inherently different composition and levels of biomass. SDI is calculated for each site and treatment as follows:

$$\mathrm{SDI}=\mathrm{TPH}\;{\left(\frac{\mathrm{QMD}}{25}\right)}^{1.605}$$

where TPH is the number of trees per hectare and QMD is the quadratic mean diameter. QMD is calculated as follows:

$$\mathrm{QMD}=\sqrt{\left(\left(\frac{\mathrm{BA}}{\mathrm{TPH}}\right)/0.00007854\right)}$$

where BA is the stand basal area per hectare. Relative density is thus calculated as follows:

$$\mathrm{RD}=\frac{\mathrm{SDI}}{{\mathrm{SDI}}_{\max}}$$

where SDI_max_ refers to the 99th percentile maximum SDI for each forest type, obtained from the literature (e.g., Schaedel, 2016; Weiskittel & Kuehne, 2019; Yang & Brandeis, 2022). A relative density of 25% of the SDI_max_ is associated with the onset of competition, 35% is associated with the lower limit of full site occupancy, and > 60% is associated with the lower limit of self‐thinning (Woodall et al., 2006).

To examine compositional diversity, Shannon's diversity index (H′DIVERSITY) was applied to species inventories for each site and treatment. Shannon's diversity was calculated for each site and treatment in terms of species and abundance (percent basal area) using the “Vegan” package in the statistical package R version 3.6.1 (Oksanen et al., 2017; R Team, 2019). Similarly, to assess the variability and the degree of diversity among management objectives applied among sites and treatments, we applied Shannon's diversity index to responses describing desired future conditions (H'DFC). In this approach, individual management objectives were treated as “species,” and their ranked importance scores (0–4) were treated as abundance values.

To assess the multivariate response of sites and treatments, nonmetric multidimensional scaling (NMS), a nonparametric ordination technique, was applied to examine gradients in variation across sites and treatments based on a matrix of global change vulnerabilities and management objectives (McCune & Grace, 2002). NMS is a rank‐based ordination technique that represents multivariate dissimilarities among samples in a reduced number of dimensions. It iteratively arranges samples so that the rank order of distances in ordination space best matches the rank order of observed dissimilarities, providing an interpretable visualization of patterns in the data and associated treatments. All NMS analyses were performed using 250 runs with real data and 250 iterations per run. Kendall correlations (tau) were calculated between a suite of hypothesized underlying predictors (NMS bi‐plots) and the resulting axis scores. A Bonferroni correction was applied to tau *p*‐values associated with correlations between values for vulnerabilities, objectives, and axis scores to correct for inflated Type 1 error. To evaluate pairwise differences among sites and treatments, Multi‐response permutation procedure (MRPP) was computed as a one‐factor permutation‐based significant test. Next, Indicator Species Analysis (ISA) was performed (based on 4999 Monte Carlo permutations) to assess significant indicators influencing sites and treatments (Dufrene & Legendre, 1997). ISA identifies species characteristic of groups by combining each species' specificity (uniqueness to a group) and fidelity (frequency within that group). Rather than using species as a predictor, we explored the role of various global change vulnerabilities and management objectives as drivers. All NMS, MRPP, and ISA were performed using the ecological statistical program PC‐ORD 6.0 (McCune & Mefford, 2011). Lastly, one and two‐way ANOVAs followed by Tukey's honestly significant difference (HSD) were used to assess differences within groups, where applicable. A significance threshold to reject the null hypothesis of no differences among groups was set for all tests at α = 0.05, and each statistical test was assessed and diagnosed to pass test assumptions including those of linearity and normality of residuals.

## RESULTS

### Starting conditions

Based on initial stand conditions prior to treatment, eight ASCC sites are dominated by gymnosperm tree species (>70%, mean Shannon's *H*′ diversity index = 0.86 ± 0.41 SE) while the remaining six sites are characterized by a mix of angiosperm and fewer gymnosperm species (hereafter referred to as angiosperm; *H*′ = 1.70 ± 0.41). Prior to harvest treatments, the mean relative density index at gymnosperm‐dominant sites was 45% (±16 SE) and at mixed angiosperm sites was 67% (±17).

Network‐wide, the role of historically important drivers of ecosystem functions differed, specifically between gymnosperm and angiosperm‐dominated sites (based on MRPP, *A* = 0.066, *p* = 0.0199; see Appendix S1: Figure S1). Among gymnosperm sites, the most important indicator based on ISA was the historical role of fire (*p* = 0.046, ranked importance = 3.6 ± 0.2, on a 0–4 scale). Additional factors that were ranked highly include the role of fire suppression (2.8 ± 0.4) and pests and pathogens (importance = 2.8 ± 0.2; Appendix S1: Table S3a). Within angiosperm sites, the most important indicator based on ISA was the historical role of ice and associated disturbances (*p* = 0.004, importance = 2.2 ± 0.2; Appendix S1: Table S3b). Additional factors that were ranked highly include legacy effects from colonial land use (agriculture, grazing, land clearing) and vegetative competition (importance = 3.0 ± 0.5, respectively).

### Global change vulnerabilities

**FIGURE 3:**
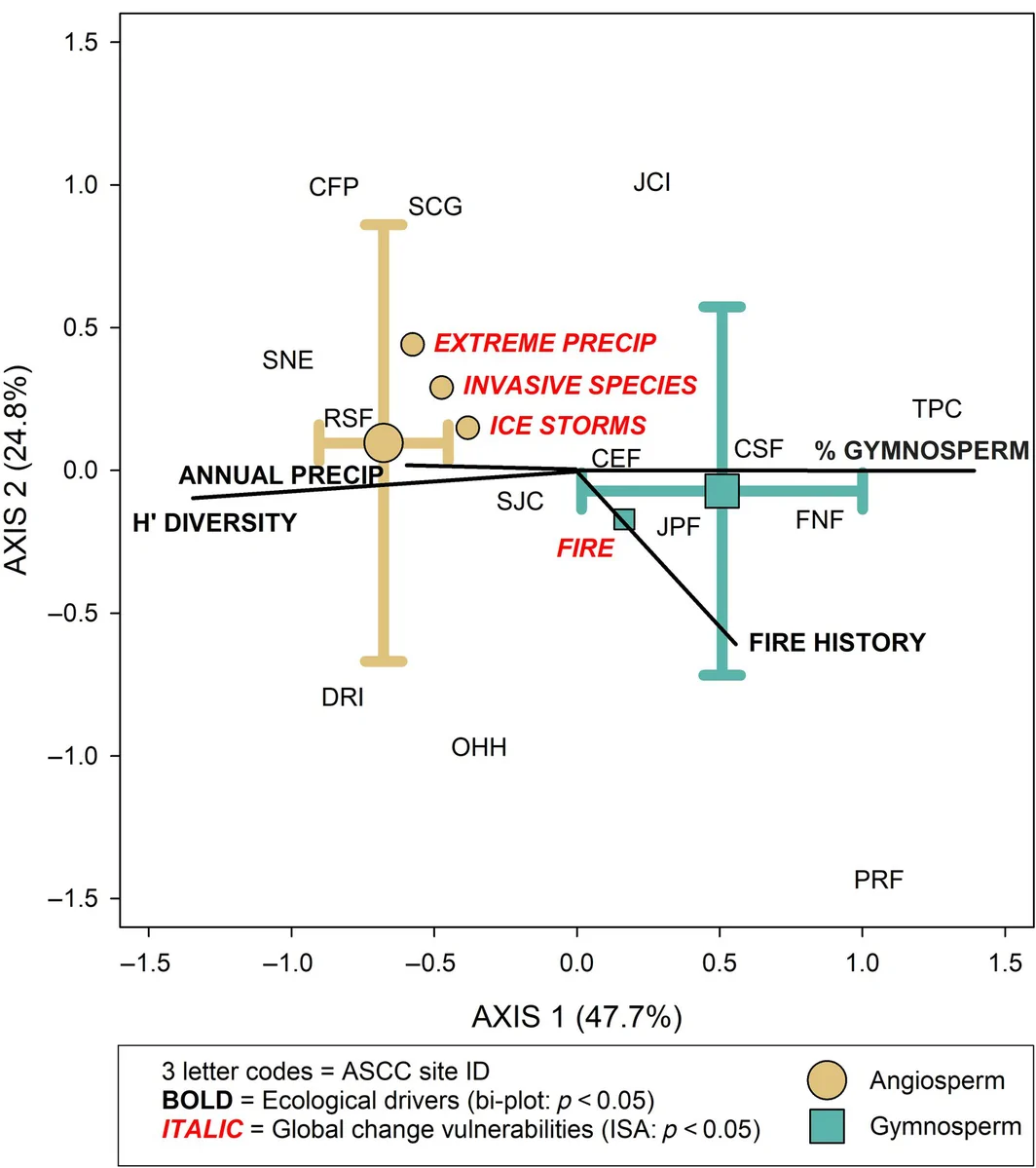
Nonmetric multidimensional scaling based on seventeen global change vulnerabilities ranked as to how important they are in terms of determining the management decisions at each ASCC site (see Appendix S1: Figure S1 for terms and raw scores). Mean axes scores (with standard error) are presented based on dominant overstory type (gymnosperm or angiosperm). Three letter site acronyms are presented in ordination space (see Table 1 for site descriptions). Joint bi‐plots (solid lines with bold, black text) are overlaid and illustrate statistically significant ecological drivers that characterize sites, where longer lines indicate stronger correlations and the orientation indicates the relationship to each axis. Significant global change vulnerabilities (scatterplot with italic, red text) based on Indicator Species Analysis (ISA) are coded by the dominant overstory group that they most strongly correspond to. See Table 2 for a list of statistically significant bi‐plot variables, Kendall tau correlations (τ), and Bonferroni corrected p‐values for each axis. Joint bi‐plot terms: % GYMNOSPERM = proportion of site overstory dominated by gymnosperm species; H′ DIVERSITY = Shannon's H′ species‐level compositional diversity of site overstory; FIRE HISTORY = the historical role of fire; ANNUAL PRECIP = Mean annual precipitation. Vulnerabilities include: fire, pests and pathogens, invasive plants, drought, extreme flooding/heavy precipitation, wind, ice storms, avalanche/landslides, shifting temperatures, altered seasonality, loss of snowpack, loss of dominant/keystone species or functional groups, species range shifts, herbivory, overabundance of species, regeneration failure, pollution.

Adaptation strategies implemented at each study site were developed to intentionally respond to local and regional climate and other global change vulnerabilities. These differences across sites are illustrated in the distribution of sites in the NMS ordination of global change vulnerabilities, which had a two‐dimensional solution with the first axis explaining 47.7% of the variation and the second axis explaining 24.8% (Figure 3, Table 2). Axis 1 ranged along measures of compositional diversity such that sites with higher Shannon's compositional diversity (H′ DIVERSITY) and lower amounts of gymnosperm overstory occupied the negative portion, with decreasing compositional diversity and increased proportion of gymnosperm dominance (% GYMNOSPERM) in the positive portion. Additionally, sites with higher mean annual precipitation (ANNUAL PRECIP) occupied the positive portion of Axis 1 and lower annual precipitation in the negative portion. Axis 2 was most strongly linked to the historical role of fire (FIRE HISTORY) on site, where sites with greater fire dynamics occupied the negative portion of the ordination space.

**TABLE 2:** Ecological drivers (bi‐plot variables) significantly correlated with axis scores from Figure 3 nonmetric multidimensional scaling ordination, with associated Kendall tau correlations (τ) and Bonferroni corrected *p*‐values.

Significant joint bi‐plot variables	Axis 1		Axis 2	
	Kendall's τ	*p*‐value	Kendall's τ	*p*‐value
Proportion gymnosperm (% GYMNOSPERM)	0.5	0.01**	0.05	0.88
Shannon's compositional diversity (H′ DIVERSITY)	−0.54	0.04*	−0.21	0.47
Mean annual precipitation (ANNUAL PRECIP)	−0.36	0.05*	0.19	0.52
Historic role of fire (FIRE HISTORY)	0.25	0.39	−0.3	0.05*

*Note*: Significance is indicated by asterisks, where * denotes *p* ≤ 0.05 and ** denotes *p* < 0.01.

Gymnosperm and angiosperm sites significantly differed in terms of the 14 global change vulnerabilities (based on MRPP; *A* = 0.064, *p* = 0.006). Based on ISA, increasing threat of fire (FIRE) was the most significant global change vulnerability indicator value among gymnosperm sites which differed significantly from angiosperm sites (*p* = 0.005), whereas flooding or heavy precipitation (EXTREME PRECIP; *p* = 0.009), ice storms (ICE STORMS; *p* = 0.011), and invasive species (INVASIVE SPECIES; *p* = 0.020) were significant vulnerability indicators of angiosperm sites (Figure 3). Although some variability still exists in terms of the ranked importance of other global change vulnerabilities, the remaining 10 vulnerability metrics did not differ among broad site types (*p* > 0.05).

### Management goals and desired future conditions

**FIGURE 4:**
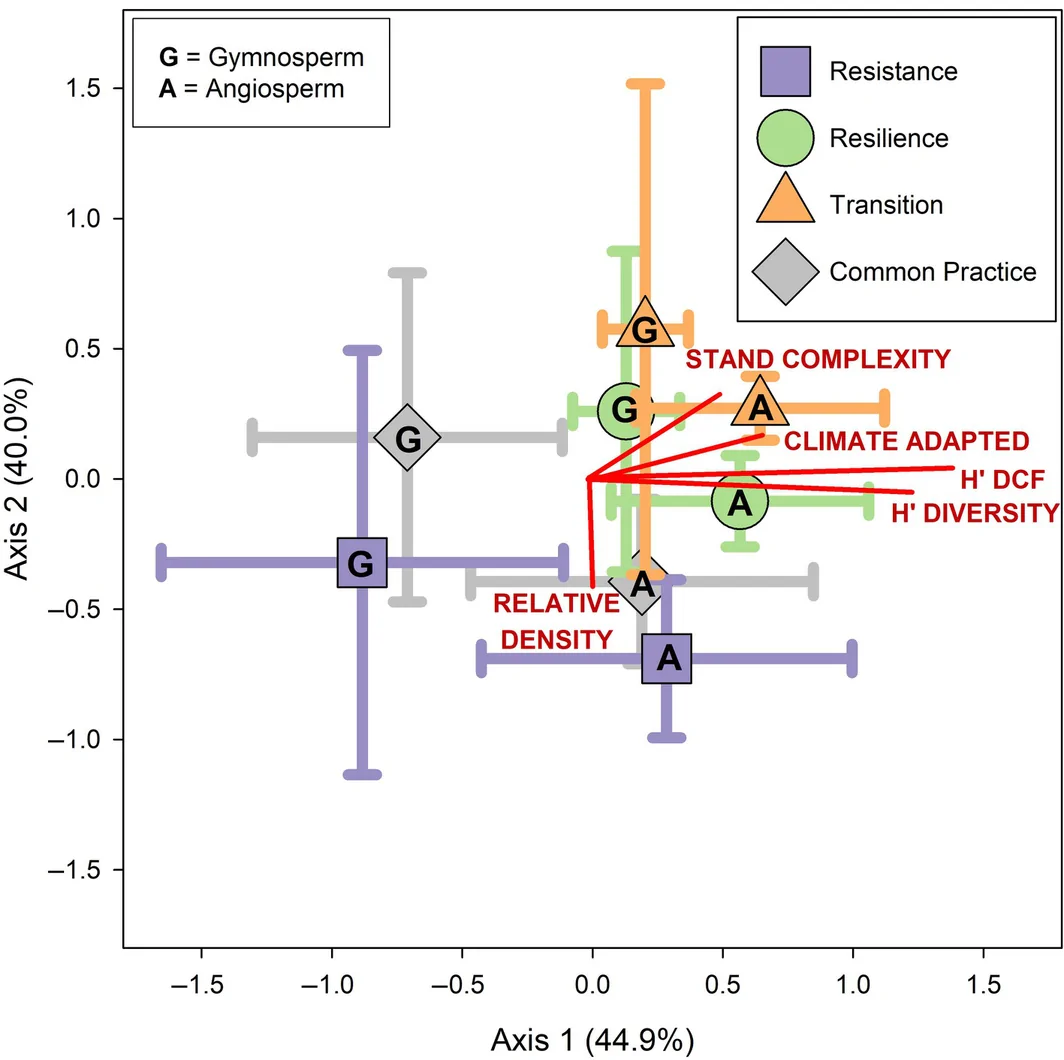
Nonmetric multidimensional scaling based on 19 forest management objectives used to develop Resistance–Resilience–Transitions treatments across the ASCC network, relative to a regional Common Practice scenario (see Figure 5 for treatment‐level scores). Mean axes scores (with standard error) are presented among groups based on treatment type and dominant overstory type (gymnosperm or angiosperm, coded “G” and “A”, respectively). Joint bi‐plots (solid lines with bold, red text) illustrate statistically significant forest management objectives, where longer lines indicate stronger correlations and the orientation indicates the relationship to each axis. See Table 3 for a list of bi‐plot variables, Kendall tau correlations (τ), and Bonferroni corrected *p*‐values for each axis. Joint bi‐plot terms are defined as: H′DFC = Shannon's diversity index (H′) applied to the desired future conditions based on nineteen management objectives; H′ DIVERSITY = Shannon's diversity index of live trees >10 cm; CLIMATE ADAPTED = future climate adaptability based on the proportion (%) of species onsite forecast to be adapted to future climatic conditions based on regional species‐distribution models; STAND COMPLEXITY = residual stand complexity, or the number of overstory conditions generated through harvesting (e.g., matrix, gap, reserve, thinning); RELATIVE DENSITY = relative density. Management objectives include: density reduction, enhance structural complexity, regenerate (natural regeneration), regenerate (artificial regeneration), maintain current species composition, diversify composition, promote future adapted species already present on site, promote future adapted species not currently present on site, enhance wildlife benefits, fire management (prescribed burning, managed wildfire, etc.), restore degraded species/ecosystems, fire suppression/reduce fuels, mitigate runoff, increase dead and downed wood, mitigate risks of invasive species, maintain stable carbon stores and accretion, promote visual aesthetics, support timber industry by promoting wood production of commercial species, support and provide recreational opportunities.

Sites implemented various forest management strategies relative to the three RRT adaptation treatments and the Common Practice scenario. These differences are illustrated in the distribution of treatments among dominant overstory groups (i.e., gymnosperm, angiosperm) in the NMS ordination of forest management goals, which had a two‐dimensional solution with the first axis explaining 44.9% of the variation and the second axis explaining 40.0% (Figure 4, Table 3). Axis 1 was principally linked to the achievement of a diversity of desired future conditions (H'DFC, based on Shannon's H′ diversity index), where sites with treatments developed with the greatest diversity in management goals occupied the positive portion of the axis. Additionally, Axis 1 was also associated with two measures of compositional diversity, where the positive portion was occupied by sites focused on treatments to increase levels of compositional diversity (H′DIVERSITY, based on Shannon's H′ diversity index) and climate adaptability (CLIMATE ADAPTED) measured in terms of the proportion of species projected to be adapted to future climatic conditions. Axis 2 was most strongly linked to the structural outcomes from treatments, where sites with treatments containing higher levels of relative density (RELATIVE DENSITY) occupied the negative portion and those with higher levels of postharvest residual stand complexity (STAND COMPLEXITY) occupied the positive portion.

**TABLE 3:** Management objectives (bi‐plot variables) significantly correlated with axis scores from Figure 4 nonmetric multidimensional scaling ordination, with associated Kendall tau correlations (τ) and Bonferroni corrected *p*‐values.

Significant joint bi‐plot variables	Axis 1		Axis 2	
	Kendall's τ	*p*‐value	Kendall's τ	*p*‐value
Shannon's compositional diversity (H′ DIVERSITY)	0.40	0.00***	0.16	0.24
Shannon's diversity of desired future conditions (H′ DFC)	0.25	0.05*	0.17	0.20
Proportion climate‐adapted species (CLIMATE ADAPTED)	0.24	0.05*	0.21	0.12
Relative density (RELATIVE DENSITY)	0.14	0.31	−0.22	0.04*
Residual stand complexity (STAND COMPLEXITY)	0.23	0.09	0.35	0.05*

*Note*: Significance is indicated by asterisks, where * denotes *p* ≤ 0.05, ** denotes *p* < 0.01, and *** denotes *p* < 0.001.

Angiosperm sites were more closely associated with greater diversity in composition and desired future conditions, as illustrated by their distribution along the positive portion of Axis 1 ordination space. These sites also maintained higher relative densities and lower stand‐level structural complexity, as linked to their position within the negative portion of Axis 2. Conversely, gymnosperm sites maintained lower levels of diversity in terms of species composition as well as desired future conditions, as illustrated by their distribution in the lower and negative portions of Axis 1 ordination space. Gymnosperm sites were also associated with lower relative density and higher stand‐level structural complexity, given that they occupy the positive portion of Axis 2.

In terms of RRT adaptation treatments, network‐wide, Resistance treatments were most closely linked to higher relative density and lower stand‐level structural complexity, due to their position in the negative portion of Axis 2 ordination space. Conversely, Resilience and Transition treatments trended in a similar direction, occupying the positive portion of both axes, which was most closely linked to reduced relative density, increased stand complexity, and increased diversity in composition and management outcomes.

Based on MRPP, the broad patterns in overstory group × treatment in Figure 4 differed significantly in terms of the 19 forest management objectives (*A* = 0.099, *p* < 0.001), with pairwise differences apparent between nearly all groups (Appendix S1: Table S4). Among RRT adaptation treatments, trends were similar between gymnosperm and angiosperm groups based on ISA, such that the most significant management objective from Resistance treatments was *maintaining current composition* (*p* = 0.0221 and 0.0006, respectively; for clarity, values not shown in Figure 4 ordination space). For Resilience treatments, *diversifying composition* (*p* = 0.0336 and 0.0384, respectively) and *enhancing structural complexity* (*p* = 0.0052 and 0.0312, respectively) were the most important indicators. Lastly, the promotion of FAM via *assisted population migration* (*p* = 0.0492 and 0.0422, respectively) and *assisted range expansion* (*p* = 0.0004 and 0.0014, respectively) were the most important indicators for Transition treatments. Among Common Practice, *supporting timber industry by promoting wood production of commercial species* was the most important indicator (*p* = 0.0312), but only among gymnosperm sites.

**FIGURE 5:**
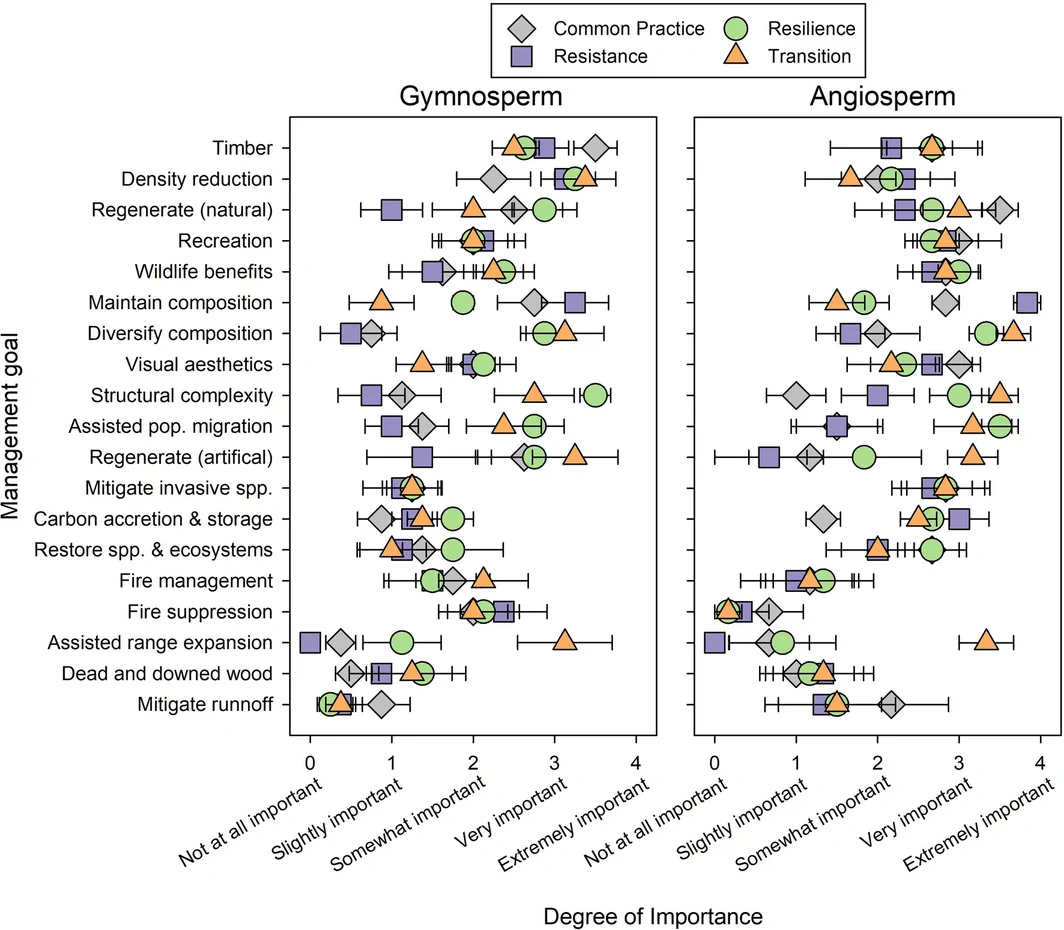
Management goals ranked in terms of degree of importance across Resistance–Resilience–Transition treatments and Common Practice among the 14ASCC sites, presented in terms of average (and SE) importance among dominant overstory types (i.e., gymnosperm, angiosperm). Management goals (*y*‐axis) are presented in ranked order highest to lowest in terms of mean degree of importance collectively across all sites network‐wide.

To more closely examine the univariate tendencies among RRT treatments and Common Practice, we illustrate the degree of importance of each management goal relative to dominant overstory types (Figure 5). Overall, the ranked importance of specific management goals among treatments was similar between broad overstory groups, although some key differences were evident. For instance, managers of gymnosperm‐dominated sites place higher importance on factors related to fire management and suppression while managers of angiosperm‐dominated sites place higher importance on mitigation of invasive pests and pathogens, carbon, and restoration of keystone species and ecosystems. Although we report key goals that differ among treatments in the previous section, which are further reinforced in Figure 5, there are notably several management goals that do not appear to deviate in importance relative to RRT treatments and Common Practice. These include but are not limited to timber products, density management, maintenance of recreational opportunities, and protection of wildlife benefits.

### Managing for complexity and diversity

**FIGURE 6:**
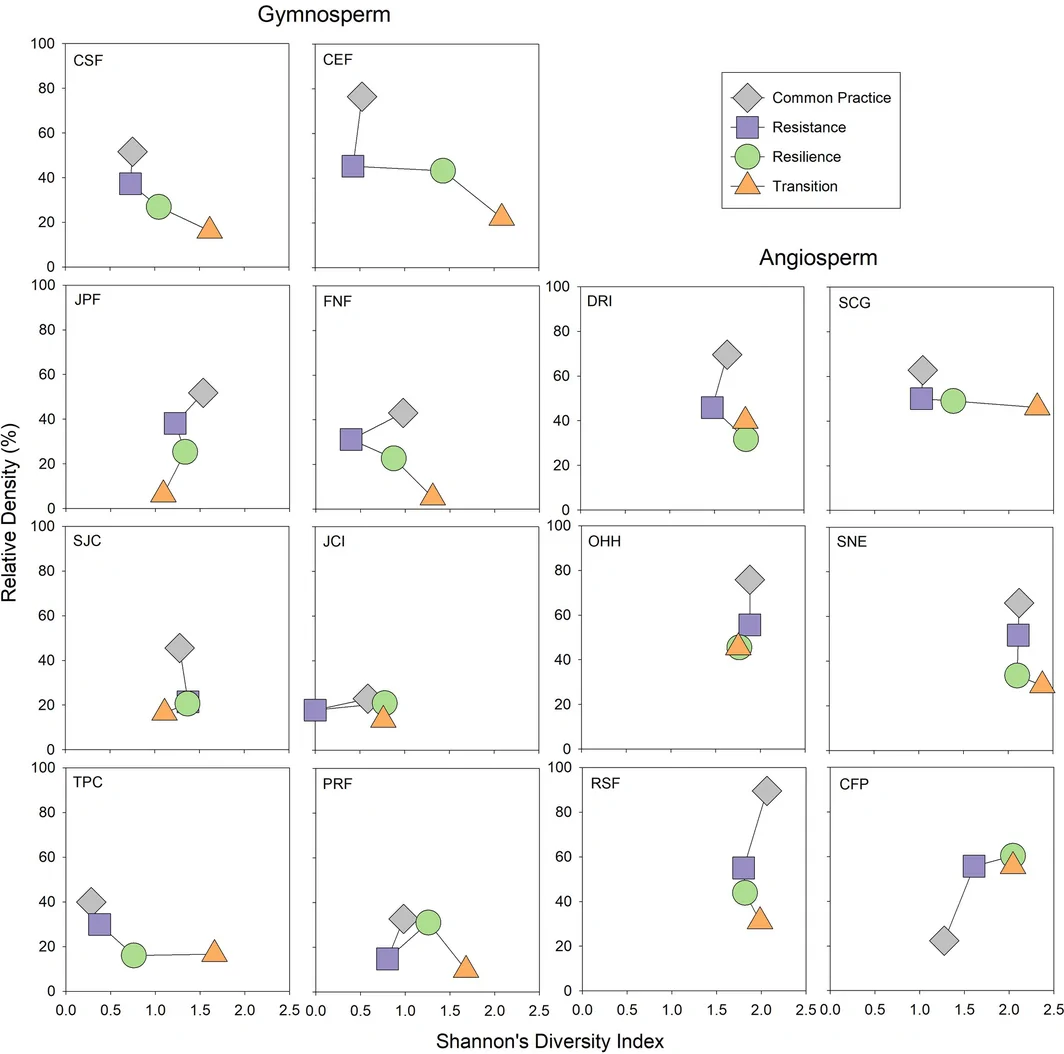
Site and treatment‐level structural complexity and compositional diversity, presented in terms of relative density (percent) and Shannon's diversity index on overstory species, respectively. Panels are separated by sites dominated by gymnosperms (left) and angiosperms (right). Three letter site acronyms are presented (see Table 1 for site descriptions).

Themes related to structural complexity and compositional diversity differ among treatments throughout the ASCC Network, yet important differences are apparent among these components when examined at the site level (Figure 6). Generally, relative density decreases while compositional diversity increases along the RRT treatment gradient, yet these trends are not always consistent or linear and vary by site. No clear trends emerge between overstory types (angiosperm and gymnosperm), potentially attributed to site‐level differences in initial conditions (e.g., stocking, diversity) and site goals or desired future conditions.

### Forest assisted migration (FAM)

Artificial regeneration and the use of FAM was a key tactic used (or expected to be used) in every site aimed at modifying species composition for climate adaptation. Although artificial regeneration was a tactic used among all treatments, the application varied among site types and objectives. FAM was overwhelmingly applied or planned to be included within Transition treatments (100% of sites) and to a lesser extent within Resilience treatments (gymnosperm = 75.0% and angiosperm = 50.0%). Only one site, PRF, employed FAM (assisted population migration) in the Resistance treatments.

Among Transition treatments, 42.4% of seedling plantings in gymnosperm‐dominated sites were classified as assisted population migration, 24.4% as assisted range expansion, and 4.1% as assisted species migration, while 11.6% of plantings are non‐FAM enrichment plantings (locally sourced seed and species). Among angiosperm‐dominated sites, 36.1% of artificial regeneration was classified as assisted population migration, 52.4% as assisted range expansion, and 0.0% as assisted species migration, while 9.4% of plantings are non‐FAM enrichment plantings. On average, plantings at gymnosperm‐dominated sites included 1.9 (0.38 SE) assisted population migration species, 1.9 (0.72) assisted range expansion species, and 0.25 (0.18) assisted species migration species, along with 0.8 (0.43) non‐FAM enrichment species. Conversely, on average plantings at angiosperm‐dominated sites included 3.2 (0.83) assisted population migration species, 4.3 (0.77) assisted range expansion species, and 0.0 (0.0) assisted species migration species, along with 1.7 (0.72) non‐FAM enrichment species.

Artificial regeneration and FAM played a smaller role in the Resilience treatment, where six gymnosperm and three angiosperm‐dominated sites employed artificial regeneration, of which 31.7 and 59.3% were assisted population migration, 41.7 and 14.8% were assisted range expansion, and 26.7 and 25.9% were non‐FAM enrichment plantings, respectively. No sites employed assisted species migration within Resilience treatments.

## DISCUSSION

Our ability to sustain ecosystem function and foster resilience will increasingly rest on our understanding of how ecosystems respond to disturbance and chronic stress under a changing climate, and how climate adaptation approaches may help shape these responses into the future (Millar et al., 2007; Seidl & Turner, 2022). As such, numerous calls have been made for operationalizing adaptation concepts by implementing and evaluating practices to better understand how to cope with change and uncertainty associated with climate change (Nagel et al., 2025; Seidl et al., 2016; St‐Laurent et al., 2021). Site characteristics, vulnerabilities to disturbance, and desired future conditions can vary considerably among forest types, such that a one‐size‐fits‐all approach to adaptation is unlikely to be an appropriate and adequate response to the pressures of global climate change. We used science coproduction to apply a common experimental design and RRT adaptation framework in what is likely the largest replicated adaptation experiment of its kind. We illustrate the trends and the utility of cross‐site inference for evaluating regional variation in the application of common adaptation strategies implemented among major forest regions in North America. The outcomes from our work highlight the role and commonalities among key forest management approaches to promote adaptation (e.g., structural complexity, compositional diversity, FAM), while also illustrating fundamental differences when localized among forest types, treatments, vulnerabilities, and desired future conditions. Despite differences in the dominant projected drivers of change (wildfire versus extreme climate or invasive pests and pathogens), adaptation actions collectively reflect management approaches centered on restoring and enhancing structural, compositional, and functional complexity, particularly for more novel regimes (i.e., Transition).

### Conditions shaping the ASCC network (Question I)

Sites within the ASCC Network represent a suite of forest types and conditions. Although some ecoregions are underrepresented at this stage, the current Network sites capture a diverse array of dominant forest types, global change vulnerabilities, and management responses. Rather than treat sites discretely, here we show that key underlying historical drivers across the Network include dominant overstory type (classified in terms of the proportion of angiosperms and gymnosperms), level of compositional diversity, aridity, and the historical role of fire. Moreover, using the overstory grouping, we show that among a matrix of 18 potential global change vulnerabilities, fire overwhelmingly remains the key vulnerability driving management responses among gymnosperm‐dominated sites, relative to the angiosperm‐dominated sites which predominantly consider invasive species, extreme precipitation, and ice storms and related winter conditions as the key vulnerabilities. This is not to say that other disturbance agents like pests and pathogens or drought are unimportant; rather, they may represent either (1) equally important vulnerabilities among sites (irrespective of overstory type), or are (2) presently considered of lesser importance when compared to other key indicators of vulnerability, with the understanding this may change in the future. The latter may also reflect a degree of uncertainty associated with global change impacts and the inability to foresee the disturbance outside of the historical range of variability (Keane et al., 2009; Landres, Morgan, & Swanson, 1999). It is possible that site managers are focused on responding to disturbance that they have already experienced or can tangibly forecast in the future as major stressors or changes, rather than anticipating novel or unforeseen disturbance associated with more extreme future range of variability (McGann et al., 2023; Seidl et al., 2016). Nevertheless, site‐level responses to these climate vulnerabilities serve as useful benchmarks to elucidate trends across the ASCC Network as well to inform broader application of climate adaptation among land managers.

### Generalized trends across the RRT adaptation framework (Question II)

#### Resistance

Network‐wide, we show generalized trends among RRT treatments. While overstory type plays an important role in differentiating treatment‐level themes, the overall trajectory of treatments follows similar pathways in ordination space. This trend is likely because the theoretical components underpinning treatments are similar, despite differences in how the tactics and actions are applied on each site. For instance, conceptually, managing for Resistance most often refers to actions that improve the defenses of the forest against anticipated change or directly defend the forest against disturbance to maintain relatively unchanged conditions relative to current state or resembling the historical range of variability. The Resistance option typically involves investing resources into maintaining what is currently on the landscape, rather than the promotion of new or novel conditions. It is likely for these reasons why Resistance treatments in our analysis can more closely resemble aspects of the Common Practice scenario, yet key differences still arise among these treatments when common practices may not be perceived as sufficient to maintain current ecological conditions given climate‐amplified stressors or other global change agents.

Based on results presented here, relative to Common Practice scenarios, in practice Resistance strategies most strongly result in modifications in forest structural components like reducing the tree density of stands via thinning to achieve an intended outcome. For instance, the application of thinning treatments in a Resistance treatment at ASCC sites like the John Prince Research Forest (JPF) in British Columbia may mitigate threats of catastrophic fire via fuels reduction treatments (Butler et al., 2013), or increase the available resources for residual trees to minimize drought and forest health impacts at the Cutfoot Experimental Forest (CEF) in Minnesota (Bottero et al., 2017; D'Amato et al., 2013), which serve as examples that may be applied to other forests or in response to various vulnerabilities. Conversely, although we show lesser emphasis placed on regeneration harvests in Resistance treatments, management decisions that favor compositional outcomes are still important, particularly in the context of ecological restoration. For instance, in more mesic, historically *Quercus* dominated angiosperm sites like the Robinson School Forest (RSF) in Kentucky or the Driftless Area (DRI) located between Iowa, Minnesota, and Wisconsin, Resistance treatments prioritize density reductions to counteract increases in tree densities and shade‐tolerant, mesophytic species due to fire suppression and restore conditions more favorable for *Quercus* sp. (Hanberry et al., 2014; Nowacki & Abrams, 2008). Although the theoretical underpinnings of Resistance are similar across these examples, the management tactics and intended outcomes differ markedly when localized and applied to different ecosystems and vulnerabilities. Nevertheless, as climate change progresses, we expect to see emerging similarity across sites in trends of increasing difficulty, effort, resources, and risk involved in maintaining relatively unchanged forest conditions (see Figure 1).

#### Resilience

Ecological resilience is an extensively studied topic, theorized under various heuristics (cf. Carpenter et al., 2001; Gunderson & Holling, 2002; Holling, 1973). Here, we describe a resilient ecosystem as one that may be impacted by disturbances but sustains multiple pathways and an ability to self‐organize in ways that can lead to an eventual recovery to a prior or desired reference condition. As with Resistance treatments, conceptually, Resilience strategies emphasize maintaining the characteristics of current forest systems; however, the latter differs somewhat by attempting to enhance ecosystem properties or pathways that support recovery from disturbance or the ability to persist with chronic stressors. Although these desired states may closely resemble that within a historical range of variability, acceptance of a wider range of outcomes is paramount to Resilience, even accounting for the potential future range of variation (see generalized distributions depicting the range of acceptable treatment outcomes in Figure 1 conceptualized diagram; Duncan et al., 2010; Seidl et al., 2016). Therefore, Resilience strategies allow for larger temporary deviations and thus a broader range of compositional and structural outcomes.

We observed network‐wide trends demonstrating that silvicultural tactics applied in Resilience treatments led to greater levels in structural complexity (measured in terms of lower relative density and higher residual stand conditions resulting from harvest patterns) as well as increased compositional diversity relative to Common Practice and Resistance treatments. Moreover, the scope of management outcomes measured in terms of the diversity in desired future conditions are also greater in Resilience treatments, relative to Resistance and Common Practice. For instance, sites like Second College Grant (SCG) in New Hampshire create a forest mosaic within Resilience treatments shaped by a combination of harvested canopy gaps and unharvested reserve areas (ranging from 0.04 to 0.1 ha) interspersed with a thinned forest matrix to produce varying habitat qualities and niche space for functionally diverse regeneration, insects and wildlife, and other ecosystem characteristics (e.g., light, moisture availability, temperatures, snow depth). Similarly, sites like San Juan National Forest (SJC) in Colorado use management tactics that create forest stands with multiple age classes, or cohorts of trees, resulting in an uneven‐aged structure and allowing for regeneration and the development of various age groups within the stand.

Regardless of the site and tactics used, the outcomes of this work support other works that suggest that ecosystem attributes and conditions that confer resilience include vegetation and physical structures surviving disturbance (Johnstone et al., 2016), structural and spatial complexity (Peck et al., 2014; Puettmann et al., 2009), as well as forest conditions that support increased compositional diversity that generate a high degree of redundancy in terms of plant functional traits (Biggs et al., 2020; Messier et al., 2019). The latter may be particularly important in the context of responding to threats from global climate change, given that ecosystem responses are more squarely associated with the depth and breadth of life history traits contained within constituent species, rather than the species identities themselves (Boisvert‐Marsh et al., 2020). Thus, we show that most Resilience strategies focus on enhancing two general stand attributes, compositional diversity and structural complexity, characteristics that are commonly heralded as key adaptation pathways strongly linked to theories of ecological resilience (Seidl et al., 2016). Ultimately, the effort, resources, and risk required to invest in creating these characteristics may increase as climate change progresses, but an outcome from Resilience treatments may result in increased levels of adaptive capacity to recover from a disturbance and maintain forest functions considering these changes.

#### Transition

Managing for Transition means intentionally facilitating change and supporting ecosystems to adaptively respond to changing and new conditions. Conceptually, the Transition option requires making an intentional, directed change to an ecosystem, which can include trying more novel approaches (i.e., FAM, significant shifts in structure) to accommodate existing and expected ecological shifts and otherwise foster resilience to a range of plausible future climate conditions. Transition strategies may represent the largest deviation from more traditional forest management practices (i.e., Common Practice) and are applied under the assumption that prevailing disturbance regimes and future climate conditions will become less suitable for current species (or genotypes) and forest structural characteristics (Rissman et al., 2018). These strategies may also be outside of the bounds of historical range of variation and aim to direct systems towards a future, more novel ecosystem state (Fisichelli et al., 2016; Millar et al., 2007; Schuurman et al., 2022). Given forest ecosystems are characterized by long‐lived, sessile plants and legacy effects of successional pathways (i.e., ecological memory; cf., Johnstone et al., 2016) the risk, efforts, and resources required to transition an ecosystem come with trade‐offs, particularly in the near term, to be able to realize a future adapted state in the long term (see conceptual diagram in Figure 1 for illustration). For instance, the planting of genotypes or species that are adapted to the climatic conditions decades from now may come at the expense of near‐term performance of those plantings (in terms of growth, survival, and other maladapted traits; Clark et al., 2022). Yet, success may be realized over the longer term via the capacity of viable future adapted plantings to sustain or augment forest function under a range of shifting climate regimes.

We show that on‐the‐ground, Transition treatments generally build on trends shaped within Resistance and Resilience treatments, with greater emphasis placed on shifts in structural and compositional outcomes. Notably, Transition treatments support the lowest levels of relative density but favor the promotion of future‐climate‐adapted species and genotypes, via creating growing space for climate‐adapted natural regeneration already onsite or through forest assisted migration (Clark et al., 2021, 2022; Holbrook & Puhlick, 2025; Muller et al., 2019). For instance, many sites like Taylor Park (TPC) in Colorado or the Petawawa Research Forest (PRF) in Ontario rely on harvest techniques that create high degrees of light and openness (via “clear cuts” with unharvested reserves; see Appendix S1: Table S1) as a first step in directing forest development on a course to more closely resemble future conditions. Likewise, sites like the Flathead National/Coram Experimental Forest (FNF) in Montana or Southern New England Exurban (SNE) located between Connecticut and Rhode Island may employ ostensibly different tactics to create openness and stand complexity (via “patch cuts” or “irregular seed tree harvests with uncut reserves”), these approaches are relativized to local ecosystems and site‐level desired future conditions. These approaches, combined with other factors such as natural regeneration response, FAM, and subsequent harvest entries, are expected over time to generate a forest structure and composition better suited to site‐level future range of variability.

Although our results indicate that management outcomes differ among all treatments and Common Practice, more commonalities exist between Common Practice and Resistance compared to Resilience and Transition. This is likely attributed to the tactics and desired future conditions of RRT treatments existing along a continuum from maintaining current conditions to actively promoting change (see St‐Laurent et al., 2021). In some ways, this bifurcated response partially aligns with other adaptation approaches that have subsequently been proposed, like the Resist‐Accept‐Direct framework (Schuurman et al., 2022, 2025), whereby Resilience and Transition treatments may “Direct” an ecosystem response in a way that acknowledges and accommodates the realities of expected ecosystem transformation. Moreover, as suggested by Rissman et al. (2018), adaptation strategies (referred to as Restoration, Persistence, and Transition‐to‐Novelty) are not mutually exclusive, such that natural resource managers may simultaneously restore one forest characteristic while at the same time transitioning other characteristics to novel conditions. Nevertheless, important differences exist among Resilience and Transition treatments in terms of theory and practice such as the inclusion of (or plans to include) FAM at 100% of Transition treatments in our study, which highlights that both future composition and structure may be quite different when compared to those conditions shaped by Resilience which are expected to fall more closely within the bounds of a historical range of variability.

### Localizing resistance‐resilience‐transition (Question III)

One of the grounding principles in the ASCC study design is that RRT treatments are localized among different ecosystems through science coproduction. Similar desired future conditions often exist among sites and treatments, yet the underlying approaches and tactics may differ based on ecosystem specific conditions and threats, supporting work put forth by Ontl et al. (2018). For instance, we show in our network‐wide analysis that components related to structural complexity and compositional diversity are key modifiers among RRT treatments. Generally, measures of structural complexity (measured in terms of relative density and residual stand conditions) and compositional diversity (measured in terms of Shannon's H′ and the proportion of species and genotypes that are future climate adapted) increase along the RRT continuum. Yet, nuance exists when localized among site‐specific conditions. For instance, sites like the Cutfoot Experimental Forest typify this trend in increasing complexity and diversity along the spectrum (see Figure 6). Here, starting conditions at this site are overstocked (elevated levels of relative density, resulting in low productivity and issues in forest health) and relatively low species richness such that treatments initially prioritize reduction in relative density for Resistance treatments, relative to Common Practice. Subsequently, Resilience and Transition treatments show increases in compositional diversity (and likely climate adaptability) with concurrent reductions in relative density. This pattern emerges because the site aims to restore a more woodland condition representative of precolonial fire exclusion which may promote greater resistance to drought and other vulnerabilities (Bottero et al., 2017; D'Amato et al., 2013).

Conversely, sites that may appear different in overstory conditions and other underlying conditions (e.g., aridity, diversity, key vulnerabilities) may share similar patterns, despite having different intended outcomes. For instance, the Ohio Hills site in Ohio (OHH; angiosperm‐dominated *Quercus*‐*Carya* forest type), The Jones Center at Ichauway in Georgia (JCI: gymnosperm‐dominated *Pinus palustris* forest type), and the John Prince Research Forest (JPF; gymnosperm‐dominated *Picea*‐*Abies* forest type) each principally exhibit reductions in relative density coupled with little change or even slightly diminished compositional diversity across the RRT spectrum. Here, the Ohio Hills site is inherently a compositionally diverse site with abundant future adapted species onsite, but historical land use has generated forest conditions in which some native species less suited to future conditions are disproportionately dominant (i.e., *Acer* sp.). Therefore, Ohio Hills prioritizes structural modifications in relative density across treatments to generate more favorable conditions for those native future adapted species already onsite, at the expense of competitive, future‐climate‐maladapted species. In contrast, at The Jones Center at Ichauway, treatment goals largely aim to restore *Pinus palustris*, via reductions in density and the proportion of other species (i.e., *P*. *elliottii*) via thinning and prescribed fire. Similarly, the emphasis on restoration of woodland structures, introduction of prescribed fire, and lower compositional diversity across treatments at the John Prince Research Forest reflects structural shifts to reduce future fire and drought vulnerability. Despite modest shifts in compositional diversity (measured in terms of Shannon's H), the John Prince Research Forest used scenario planning as part of the co‐development process which also resulted in the selection of reforestation tactics that favor both a warm‐wet future and a warm‐dry future.

Finally, there are sites that follow alternative trends to generate adaptative conditions. For instance, Crosby Farm Regional Park (CFP) in Minnesota is a low‐density riparian forest with vulnerable conditions shaped by species specific invasive insects and pathogens (e.g., emerald ash borer (*Agrilus planipennis*) and Dutch elm disease (*Ophiostoma novo‐ulmi*)). Given these starting conditions, Crosby Farm Regional Park prioritizes increasing relative density among all RRT treatments, via artificial regeneration to reforest the site. Although compositional diversity increases among treatments (attributed to different selections of planting stock), the key driver on this site is to reforest a degraded site.

### The role of Forest assisted migration (Question IV)

The application of FAM in the ASCC Network increases along the RRT spectrum and is disproportionately applied as a tactic in the Transition treatments, supporting Palik et al. (2022) who frame FAM within the RRT framework (e.g., Resistance: no FAM; Resilience: limited FAM, but mostly assisted population migration; Transition: stronger reliance of FAM). Although examples exist within Resilience, these instances are most common in forested regions where silvicultural systems rely on tree planting to obtain desired outcomes in terms of regeneration. These systems are commonly gymnosperm‐dominated with lower inherent species diversity, where the use of diversified, improved, or disease‐resistant genotypes of local species can be more common (Schmidtling, 2001). Therefore, the inclusion of climate‐adapted genotypes via assisted population migration plantings may represent conditions still characterized by Resilience treatments and capture conditions in which Rissman et al. (2018) refer to as “Directed Transformation”.

All sites employed (or plan to employ) some type of FAM within Transition treatments, specifically assisted population migration and assisted range expansion, highlighting the role that this tool plays in forest management aimed at transitioning ecosystems into a novel future state. Despite this trend, differences emerge when localized to the site level. For instance, many angiosperm‐dominated sites plant a greater number of FAM species which reflect the inherently higher levels of diversity in those ecosystems. Regardless of endogenous levels of diversity, sites reported that considerable attention was paid to the selection of plant functional traits, and functional redundancy of climate‐adapted species to serve as potential replacements for climate maladapted species. The incorporation of multiple climate‐adapted species that confer critical functional attributes is an important bet‐hedging approach to sustaining key ecosystem functions and/or promoting adaptive capacity in a transitioned system (Aubin et al., 2016; Boisvert‐Marsh et al., 2020), reflective of the insurance hypothesis (Yachi & Loreau, 1999). Lastly, given that FAM has been hotly debated due to the potential risks associated with invasion and unintended consequences (Hewitt et al., 2011; Palik et al., 2022; Pedlar et al., 2012), it is important to note that very few sites reported using assisted species migration (the interregional, transcontinental, and intercontinental movement of a species well beyond areas accessible via natural dispersal). The general consistency in the application of FAM types in Transition treatments across the ASCC Network (i.e., primary reliance on assisted population migration and assisted range expansion) highlights that decision makers clearly differentiate among FAM types and their appropriateness for meeting adaptation goals.

## LESSONS LEARNED

After over a decade of development and implementation of the ASCC Network, key lessons have emerged in terms of how the application of the RRT framework that present clear advancements in the translation of adaptation theory into practice. For instance, we show consistent reliance on biologically and ecologically relevant management tactics focused on a core suite of adaptation pathways principally linked to modifications to (1) stand structural characteristics (measured here in terms of relative density and residual stand complexity), (2) species composition (measured here in terms of Shannon's diversity), and (3) the strategic promotion of climate‐adapted species and genotypes (measured here in terms of natural and artificial regeneration (i.e., [FAM]). While these metrics provide a concrete basis for measuring adaptation, other measures linked to fostering adaptive capacity may serve as valuable surrogates (i.e., functional diversity). Together, they point to key and tangible targets that foresters and other resources managers are already familiar with that can be applied to promote and evaluate varying degrees of adaptation.

Another key lesson is that network‐wide patterns confirm theoretical expectations such that greater structural and compositional diversity and the use of FAM broadly increase along the RRT continuum. Nevertheless, this work underscores the importance of regional variation and flexibility by demonstrating that dominant overstory type, ecological and disturbance history, and locally impactful vulnerabilities (e.g., fire vs. extreme precipitation vs. invasive pests and pathogens) strongly shape how similar concepts are operationalized on the ground. The case studies we provide highlight that there are meaningful departures in these network‐level trends driven by present day local conditions, future threats, restoration needs, or opportunities. For instance, forests vulnerable to wildfire may prioritize density reductions to shift towards woodland structures, whereas a degraded riparian forest may prioritize reforestation and increasing stocking and diversity, and a species‐rich forest in areas less fire prone may emphasize structural adjustment over further diversification. Collectively, these results provide concrete, place‐based examples that managers can use to calibrate expectations, anticipate trade‐offs, and incorporate RRT strategies into forest management plans.

## CONCLUSION

In this synthesis, we provide experimental results of cross‐regional consistencies as well as regional variation in how forest management for adaptation is translated into on‐the‐ground practices. Nevertheless, forest management for adaptation is unavoidably iterative (see depiction in Figure 1). The ASCC sites and treatments were designed around the “best available” understanding of vulnerabilities and tools at specific points in time, yet emerging threats (e.g., novel pests), evolving acceptance and capacity for practices (e.g., FAM), or shifting social and policy priorities may alter how treatments are designed, interpreted, or maintained to achieve desired future conditions. As Network sites are monitored and iteratively adjusted over time through subsequent harvest entries, the integration of new tools, technologies, and world views must be incorporated to address new challenges of the moment. The ASCC Network provides a consistent framework to evaluate a wide range of ecological factors that should accommodate these changes in climate, society, and technology as they develop over time. The Network is a unique combination of placed‐based, operational‐scale forest adaptation treatments, and rigorous empirical experimentation that has already yielded numerous early insights and continues to provide evolving, real‐world demonstrations of adaptation on‐the‐ground that can be used to inform and refine management to better achieve the values our forest ecosystems provide in the face of a changing climate into the future.

## CONFLICT OF INTEREST STATEMENT

The authors declare no conflicts of interest.

## Supporting information


Appendix S1.

## Data Availability

The datasets generated for this study can be found at Figshare.com. See (Clark et al., 2026).
