# Supplementary material for: Adaptive silviculture for climate change in North American forests: Operationalizing the resistance–resilience–transition framework
Source: Ecol Appl. 2026 Sep 20;36(6):e70302. doi: 10.1002/eap.70302 (PMC13590238; doi:10.1002/eap.70302)

Appendix S1

Adaptive silviculture for climate change in North American forests: Operationalizing the resistance–resilience–transition framework

Peter W. Clark, Anthony W. D’Amato, Linda Nagel, Brian J. Palik, Mike A. Battaglia, Maria K.

Janowiak, Courtney L. Peterson, Christopher W. Swanston, Maria R. Vicini

Ecological Applications

Table S1: Silvicultural tactics used at each study site across the resistance–resilience–transition spectrum along with Common Regional Practice.

FAM Abbreviations: APM = assisted population migration, ARE = assisted range expansion, ASM = assisted species migration

Site	COMMON REGIONAL PRACTICE– management typical to regional forests	RESISTANCE – maintain relatively unchanged conditions	RESILIENCE – allow some change, eventual return to reference	TRANSITION – facilitate change, encourage adaptive response
**Cutfoot Experimental Forest (*CEF*)**, Minnesota, USA	Row or free thinning to moderate to high relative density, followed by clearcutting and planting of red pine.	Uniform (free) thin to low relative density (B-line).	Variable density thin (i.e., skips and gaps), with thinned matrix (as in Resistance). Planting of native species/genotypes and APM in gaps.	Expanding gap, irregular shelterwood. Stand-wide planting of native species/genotypes, APM, ARE, and ASM
**Crosby Farm Regional Park (*CFP*)**, Minnesota, USA	No overstory removal with no to limited underplanting of native species/genotypes protected by mesh tubes	All trees removed within 0.04 ha gaps. Planting of native species/genotypes protected by fencing	All trees removed within 0.04 ha gaps. Planting of native species/genotypes, APM, and ARE protected by fencing	All trees removed within 0.04 ha gaps. Planting of native species/genotypes, APM, and ARE protected by fencing
**Colorado State Forest (*CSF*)**, Colorado, USA	Group selection, thin from below (wildfire mitigation), single-tree selection	Free thin (Sanitation Dwarf Mistletoe)	Group selection (0.2 ac to 0.8 ha); matrix thin. Planting of APM and ARE	Group selection (0.2 ac to 1.6 ha); Planting of APM and ARE
**Driftless Area (*DRI*)**, Iowa, Minnesota, and Wisconsin, USA	Free thinning or thin from above followed by either conventional shelterwood or clearcutting; planting is common; high-grading is common	Free thin to ~B-line; midstory removal; prescribed fire for understory maintenance	Continuous cover irregular shelterwood; midstory removal; prescribed fire. Planting of native species/genotypes and APM	Clearcut with reserves. Planting of native species/genotypes, APM, and ARE,
**Flathead National/ Coram Experimental Forest (*FNF*)**, Montana, USA	Thinning from below to moderate relative density followed by seed tree or shelterwood harvests with planting where needed.	Commercial thinning from below	Group selection with reserves, thinning from below in the matrix. Planting of native species/genotypes	Irregular seed tree with reserves. Planting of native species/genotypes and APM
**Jones Center at Ichauway (*JCI*)**, Georgia, USA	Commercial thinning of southern pines, followed by clearcutting, site preparation, and planting.	All hardwoods cut and herbicide. Pines other than longleaf removed unless needed to maintain fuel continuity. Prescribed fire.	Mesic oaks and fleshy-fruited hardwoods cut and herbicide. Variable density thinning some dense patches of pine. Prescribed fire.	Variable density thinning across stands. Mesic oaks and fleshy-fruited hardwoods cut and herbicide. Prescribed fire. Planting of APM.
**John Prince Research Forest (*JPF*)**, British Columbia, CA	Clearcut with retention	Hybrid group selection. Planting of native species/genotypes	Hybrid group selection with variable density thinned matrix. Planting of APM	Shelterwood. Planting of APM and ARE
**Ohio Hills (*OHH*)**, Ohio, USA	Free thinning followed by regeneration methods – shelterwood, clearcut, and deferment	Thin from below, unharvested reserves, and prescribed fire	Thin from below, unharvested reserves, expanding gap irregular shelterwood, and prescribed fire	Thin from below, unharvested reserves, expanding gap irregular shelterwood, and prescribed fire. Planting of APM and ARE
**Petawawa Research Forest (*PRF*)**, Ontario, CA	2- or 3-cut uniform shelterwood. Planting of native species/genotypes	2-cut uniform shelterwood. Planting of native species/genotypes and APM	Expanding gap irregular shelterwood. Planting of APM	Clearcut with seed tree. Planting of native species/genotypes, APM and ARE
**Robinson School Forest (*RSF*)**, Kentucky, USA	Shelterwood treatment and subsequent treatment/removal of undesirable midstory to favor oak	2- or 3-step shelterwood	Extended irregular shelterwood	Variable retention harvest. Planting of native species/genotypes, APM, and ARE
**Second College Grant (*SCG*)**, New Hampshire, USA	Single-tree selection with occasional group selection	Single-tree selection	Hybrid single-tree selection & group selection (variable density thinning). Gaps and unharvested reserves up to 0.1 ha	Continuous cover irregular shelterwood (variable density thin). Gaps up to 0.4 ha. Planting of APM and ARE in gaps, 50% browse protection & competition control
**San Juan National Forest (*SJC*)**, Colorado, USA	Improvement/sanitation cuts; Single-tree selection;	Free thin (even-spacing)	Variable density thinning	Variable retention harvest. Optional planting of APM, if natural regeneration fails
**Southern New England Exurban (*SNE*)**, Connecticut and Rhode Island, USA	"Highgrade" single tree selection (non-silvicultural extractive harvest) and/or shelterwood (silvicultural harvest to regenerate oak)	Prep shelterwood cut	Small patch cuts/group selection with reduction of mid-canopy shade. Limited planting of native and APM species.	Larger patch cuts with elimination of mid-canopy shade. Limited planting of APM and ARE species.
**Taylor Park (*TPC*)**, Colorado, USA	Clearcut/patch cuts; thin from below; sanitation (dwarf mistletoe)	Commercial and precommercial thin (uniform).	2-step group shelterwood with groups 0.1 to 2.0 ha; variable density thinned matrix. Planting of native species/genotypes, APM, and ARE	Clearcut with reserves followed by broadcast burn or pile burns. Planting of native species/genotypes APM and ARE

Section S1: Mixed-methods data collection and survey prompts used to generate a standardized dataset among ASCC sites administered using a Qualtrics survey instrument. Each site populated responses to the following prompts. For clarity, duplicate questions used across all treatments are only presented once and noted as such.

What is the name of your ASCC site?______________________

THE FOLLOWING QUESTIONS AIM TO CAPTURE INFORMATION ABOUT THE STARTING CONDITIONS, VULNERABILITIES, AND OTHER FACTORS THAT INFLUENCE THE DEVELOPMENT AND MANAGEMENT AT YOUR SITE.

What are the starting conditions at your ASCC site in terms of stocking, expressed in terms of mean basal area (m2/Ha) and trees per hectare (approximately >4in or 10cm)?

basal area (m2/Ha)______________
trees per hectare________________

What are the tree species currently on site expressed in terms of percent (%) abundance based on basal area (total should equal 100).

Species name	Percent abundance
________________	___________________
________________	___________________
________________	___________________
________________	___________________
________________	___________________
________________	___________________
________________	___________________
________________	___________________
________________	___________________
________________	___________________
________________	___________________

HISTORICAL CONDITIONS, VULNERABILITIES, AND MANAGEMENT DECISIONS

Rank the following factors (unrelated to global climate change) that historically shaped ecosystem dynamics at your ASCC site in terms of:

(0) Not at all influential
(1) Slightly influential
(2) Somewhat influential
(3) Very influential
(4) Extremely influential

Fire_____
Pests and pathogens_____
Drought_____
Flooding_____
Vegetative competition_____
Indigenous management_____
Colonial land use (agriculture, grazing, land clearing) _____
Wind_____
Ice_____
Avalanche/landslides_____
Fire suppression_____
Other (Optional write in response) _____

Rank the following vulnerabilities as to how important they are in terms of determining the management decisions at your ASCC site in terms of:

(0) Not at all influential
(1) Slightly influential
(2) Somewhat influential
(3) Very influential
(4) Extremely influential

Fire_____
Pests and pathogens_____
Invasive plants_____
Drought_____
Flooding / heavy precipitation_____
Wind_____
Ice_____
Avalanche/landslides_____

Shifting temperatures_____
Altered seasonality_____
Loss of snowpack_____
Loss of dominant/keystone species or functional groups_____
Species range shifts_____
Herbivory_____
Overabundance of species_____
Regeneration failure_____
Pollution_____
Other (Optional write in response) _____

Rank the following management goals as to how they relate to common silvicultural practices "typically" used in your region or in forest types comparable to your ASCC site. (This is analogous to a "business-as-usual" scenario.) Use the following ranking system:

(0) Not at all influential
(1) Slightly influential
(2) Somewhat influential
(3) Very influential
(4) Extremely influential

Density reduction_____
Enhance structural complexity_____
Regenerate (natural regeneration) _____
Regenerate(artificial regeneration) _____
Maintain current species composition_____
Diversify composition_____
Promote future adapted species already present on-site_____
Promote future adapted species not currently present on-site_____
Enhance wildlife benefits_____
Fire management(Rx burning, managed wildfire, etc.) _____
Restore degraded species /ecosystems_____
Fire suppression /reduce fuels_____
Mitigate runoff_____
Increase dead and downed wood_____
Mitigate risks of invasive species_____
Maintain stable carbon stores and accretion_____
Promote visual aesthetics_____
Support timber industry by promoting wood production of commercial species_____
Support and provide recreational opportunities_____

THE FOLLOWING QUESTIONS ARE REPEATED ACROSS RESISTANCE, RESILIENCE, AND TRANSITION TREATMENTS

[This question is replicated for each RRT treatment]

Rank the following management goals in terms of how important they are for each of the RESISTANCE, RESILEINCE, AND TRANSITION TREATMENTS at your site. Use the following ranking system:

(0) Not at all influential
(1) Slightly influential
(2) Somewhat influential
(3) Very influential
(4) Extremely influential

Density reduction_____
Enhance structural complexity_____
Regenerate (natural regeneration) _____
Regenerate(artificial regeneration) _____
Maintain current species composition_____
Diversify composition_____
Promote future adapted species already present on-site_____
Promote future adapted species not currently present on-site_____
Enhance wildlife benefits_____
Fire management(Rx burning, managed wildfire, etc.) _____
Restore degraded species /ecosystems_____
Fire suppression /reduce fuels_____
Mitigate runoff_____
Increase dead and downed wood_____
Mitigate risks of invasive species_____
Maintain stable carbon stores and accretion_____
Promote visual aesthetics_____
Support timber industry by promoting wood production of commercial species_____
Support and provide recreational opportunities_____

[This question is replicated for each RRT treatment]

What are the conditions (post-treatment) for each of the RESISTANCE, RESILEINCE, AND TRANSITION TREATMENTS in terms of stocking, expressed in basal area (m2/Ha) and trees per hectare (>4in or 10cm)?

Given that some treatments create varying harvest conditions (e.g., skip, gap, matrix) with different levels of stocking, please describe each harvest condition and report stocking for each of these conditions as well as the percent of the treatment unit area that they encompass.

Harvest condition type	Percent of treatment area	BA m2/Ha	TPHa
____________________	__________________	__________	__________
____________________	__________________	__________	__________
____________________	__________________	__________	__________
____________________	__________________	__________	__________
____________________	__________________	__________	__________

[This question is replicated for each RRT treatment]

What are the conditions (*post-treatment*) for each of the RESISTANCE, RESILEINCE, AND TRANSITION TREATMENTS expressed in terms of species composition?

1. Record the species name.
2. Report the percent (%) abundance (total must equal 100).
3. Report the current presence of each species relative to the present day, in terms of the following categories:

   Naturally regenerated options

   A) currently onsite, naturally regenerated, and forecasted to be future adapted (no change or some increase in habitat suitability)

   B) currently onsite, naturally regenerated, and not forecasted to be future adapted (some decrease in habitat suitability)

   Artificially regenerated options (species range encompasses site)

   C) currently onsite/within species range, artificially regenerated with local seed source, and forecasted to be future adapted (no change or some increase in habitat suitability)

   D) currently onsite/within species range, artificially regenerated with local seed source, and not forecasted to be future adapted (some decrease in habitat suitability)

   E) currently onsite/within species range but artificially regenerated using a future adapted seed source/genotype (e.g., from warmer, southern, or lower elevation site)

   Artificially regenerated options (species range does not encompass site)

   F) not within current/historical species range, but the range is nearby and expected to shift under climate change to increase habitat suitability at the site. Artificially regenerated

   G) not within current/historical species range. The species' range is nowhere close and the species would never naturally migrate to site (e.g., transcontinental shift). Artificially regenerated

   H) other option (briefly explain in cell)

Species name	Percent abundance (%)	Current presence (A-G)

[This question is replicated for each RRT treatment]

Report the tree species and sources you are planting (or plan to plant) for each of the RESISTANCE, RESILEINCE, AND TRANSITION TREATMENTS

1) Write the species name
2) Report if the seed source is:
    a) local to the site (*within <100km*)
    b) short transfer distance from a southern, warmer, lower elevation, or otherwise future-climate adapted seed zone (*approx. 100-200 km from site*)
    c) long transfer distance from a southern, warmer, lower elevation, or otherwise future-climate adapted seed zone (*approx. 200-800 km from site*)
    d) exotic, transcontinental, or non-native species that would never realistically get to site without assistance.
3) Report the forest assisted migration (FAM) type for each species. (For clarification about FAM types, visit: https://www.fs.usda.gov/ccrc/topics/assistedmigration):
    a) assisted population enrichment (PE): enrichment of a species already onsite using climate adapted seed/genotype from a southern/warmer/lower elevation environment
    b) assisted range expansion (RE): movement of a new species not already onsite/not within the species' current range but its range is expected to shift and increase habitat suitability at the site over long time scales
    c) assisted species migration (SM): transcontinental movement of exotic/non-native species never expected to naturally migrate to site due to a natural migration barrier
    d) non FAM enrichment planting: local genotype planted to enrich species already onsite.

Species name	Source	FAM type

Table S2: List of categories and associated factors that sites scored relative to the degree of influence or importance.

Category	Factor
10 historically important environmental conditions	a) fire, b) pests and pathogens, c) drought, d) flooding, e) wind, f) ice, g) vegetative competition, h) indigenous management, i) colonial land use (agriculture, grazing, land clearing), and j) fire suppression.
18 global change vulnerabilities	a) fire, b) novel pests and pathogens, c) invasive plants, d) drought, e) flooding/heavy precipitation, f) wind, g) ice, h) avalanche/landslides, i) shifting temperatures, j) altered seasonality, k) loss of snowpack, l) loss of dominant/keystone species or functional groups, m) species range shifts, n) herbivory, o) overabundance of species, p) regeneration failure, and q) pollution.
19 management objectives	a) density reduction, b) enhance structural complexity, c) regenerate (natural regeneration), d) regenerate (artificial regeneration), e) maintain current species composition, f) diversify composition, g) use assisted population migration (APM) to promote future adapted species already present on-site, h) use assisted range expansion (ARE) promote future adapted species not currently present on-site, i) enhance wildlife benefits, j) fire management (prescribed burning, managed wildfire, etc.), k) restore degraded species/ecosystems, l) fire suppression/reduce fuels, m) mitigate runoff, n) increase dead and downed wood, o) mitigate risks of invasive species, p) maintain stable carbon stores and accretion, q) promote visual aesthetics, r) support timber industry by promoting wood production of commercial species, and s) support and provide recreational opportunities.

Table S3: Ranked importance of historical factors on (a) gymnosperm amd (b) angiosperm sites. Ranked scores based on Likert scores (0 = Not at all important, 4 = Extremely important). Pairwise factors that do not differ at p>0.05 are assigned the same letter, based on Tukey Honesty Significance.

Historically important driver terms: **COLON** = Colonial land use (agriculture, grazing, land clearing), **DROUGHT** = Drought, **FIRE** = Fire, **FIRESUP** = Fire suppression, **FLOOD** = Flooding events**,** **ICE** = Ice damage, **INDIGEN**= Indigenous management, **PESTPATHS** = Pests and pathogens, **VEGCOMP** = Vegetative competition, **WIND** = Wind events (e.g., hurricanes, tornados)

**Term**	**mean**	**se**	**Significance groups (Tukey HSD)**
**(a) Gymnosperm**			
FIRE	3.6	0.2	a
FIRESUP	2.8	0.4	ab
PESTSPATHS	2.8	0.2	ab
VEGCOMP	2.6	0.3	ab
WIND	2.3	0.2	abc
COLON	1.8	0.6	bcd
INDIG	1.8	0.5	bcd
DROUGHT	1.5	0.2	bcd
ICE	0.9	0.2	cd
FLOOD	0.3	0.2	d
**(b) Angiosperm**			
COLON	3	0.5	a
VEGCOMP	3	0.3	a
PESTSPATHS	2.8	0.3	a
INDIG	2.7	0.6	a
DROUGHT	2.5	0.5	a
FIRESUP	2.5	0.7	a
WIND	2.3	0.4	a
FIRE	2.2	0.7	a
ICE	2.2	0.2	a
FLOOD	1.2	0.6	a

Table S4: MRPP pairwise differences among treatments (RRT and Common Practice) and broad overstory groups (angiosperm and gymnosperm) based on 19 management objectives, as depicted in Figure 4.

**Pairwise group comparisons (treatment x broad overstory groups)**			**T**	**A**	**P-value**
Common Practice (Gymnosperm)	vs.	Transition (Angiosperm)	-5.524	0.1591	**0.0004******
Resistance (Gymnosperm)	vs.	Transition (Angiosperm)	-6.327	0.2015	**0.0005******
Resistance (Angiosperm)	vs.	Transition (Gymnosperm)	-5.514	0.1595	**0.0009******
Resistance (Angiosperm)	vs.	Resilience (Gymnosperm)	-4.666	0.11	**0.0009******
Resistance (Gymnosperm)	vs.	Transition (Gymnosperm)	-4.909	0.1204	**0.001******
Resistance (Gymnosperm)	vs.	Resilience (Angiosperm)	-5.458	0.1527	**0.001******
Common Practice (Angiosperm)	vs.	Transition (Gymnosperm)	-5.243	0.1489	**0.0013*****
Common Practice (Gymnosperm)	vs.	Resilience (Angiosperm)	-4.652	0.1288	**0.0013*****
Common Practice (Angiosperm)	vs.	Resilience (Gymnosperm)	-4.468	0.1131	**0.0015*****
Resistance (Gymnosperm)	vs.	Resilience (Gymnosperm)	-4.183	0.1029	**0.002*****
Common Practice (Gymnosperm)	vs.	Transition (Gymnosperm)	-4.013	0.0921	**0.0025*****
Resistance (Angiosperm)	vs.	Transition (Angiosperm)	-3.638	0.1262	**0.0032*****
Common Practice (Gymnosperm)	vs.	Resilience (Gymnosperm)	-3.562	0.0811	**0.0048*****
Common Practice (Gymnosperm)	vs.	Resistance (Angiosperm)	-3.432	0.0828	**0.0051*****
Common Practice (Angiosperm)	vs.	Resistance (Gymnosperm)	-3.566	0.083	**0.0052*****
Resilience (Angiosperm)	vs.	Transition (Gymnosperm)	-3.647	0.095	**0.0074*****
Common Practice (Angiosperm)	vs.	Transition (Angiosperm)	-3.233	0.1222	**0.0097*****
Resistance (Angiosperm)	vs.	Resistance (Gymnosperm)	-2.683	0.0631	**0.016***
Resilience (Gymnosperm)	vs.	Transition (Angiosperm)	-2.432	0.0553	**0.0203***
Transition (Angiosperm)	vs.	Transition (Gymnosperm)	-2.253	0.0493	**0.0288***
Common Practice (Angiosperm)	vs.	Common Practice (Gymnosperm)	-2.086	0.0523	**0.0389***
Common Practice (Angiosperm)	vs.	Resilience (Angiosperm)	-1.542	0.0565	0.0724
Resilience (Angiosperm)	vs.	Resilience (Gymnosperm)	-1.432	0.0314	0.089
Resistance (Angiosperm)	vs.	Resilience (Angiosperm)	-1.192	0.0388	0.1205
Resilience (Gymnosperm)	vs.	Transition (Gymnosperm)	-0.474	0.0127	0.2508
Common Practice (Angiosperm)	vs.	Resistance (Angiosperm)	-0.129	0.0038	0.4027
Common Practice (Gymnosperm)	vs.	Resistance (Gymnosperm)	0.224	-0.005	0.5151
Resilience (Angiosperm)	vs.	Transition (Angiosperm)	0.1949	-0.008	0.5237

Figure S1: The importance of various historically important ecological drivers and global change vulnerabilities among ASCC sites. Importance is assessed on a 0-4 scale, where 0= not at all important, 1 = slightly important, 2= somewhat important, 3 = very important, 4 = extremely important.

Historically important driver terms: **COLON** = Colonial land use (agriculture, grazing, land clearing), **DROUGHT** = Drought, **FIRE** = Fire, **FIRESUP** = Fire suppression, **FLOOD** = Flooding events, **ICE** = Ice damage, **INDIGEN**= Indigenous management, **PESTPATHS** = Pests and pathogens, **VEGCOMP** = Vegetative competition, **WIND** = Wind events (e.g., hurricanes, tornados)

Global Change Vulnerability terms: **ATLSSON** = Altered seasonality, **BROWSE** = Browse and herbivory, **DROUGHT** = Drought, **EXPCP** = Flooding / heavy precipitation, **FIRE** = Fire, **ICE** = Ice events, **INVPLANTS** = Invasive plants, **KEYSTONE** = Loss of dominant /keystone species or functional groups, **LOSSSNOW** = Loss of snowpack, **OVERABUND** = Overabundance of species, **PESTPATHS** = Pests and pathogens, **POLLUT** = Pollution, **REGNFAIL** = Regeneration failure, **SPRANGE** = Species range shifts, **SHIFTTMP** = Shifting temperatures, **WIND** = Wind events (e.g., hurricanes, tornados)

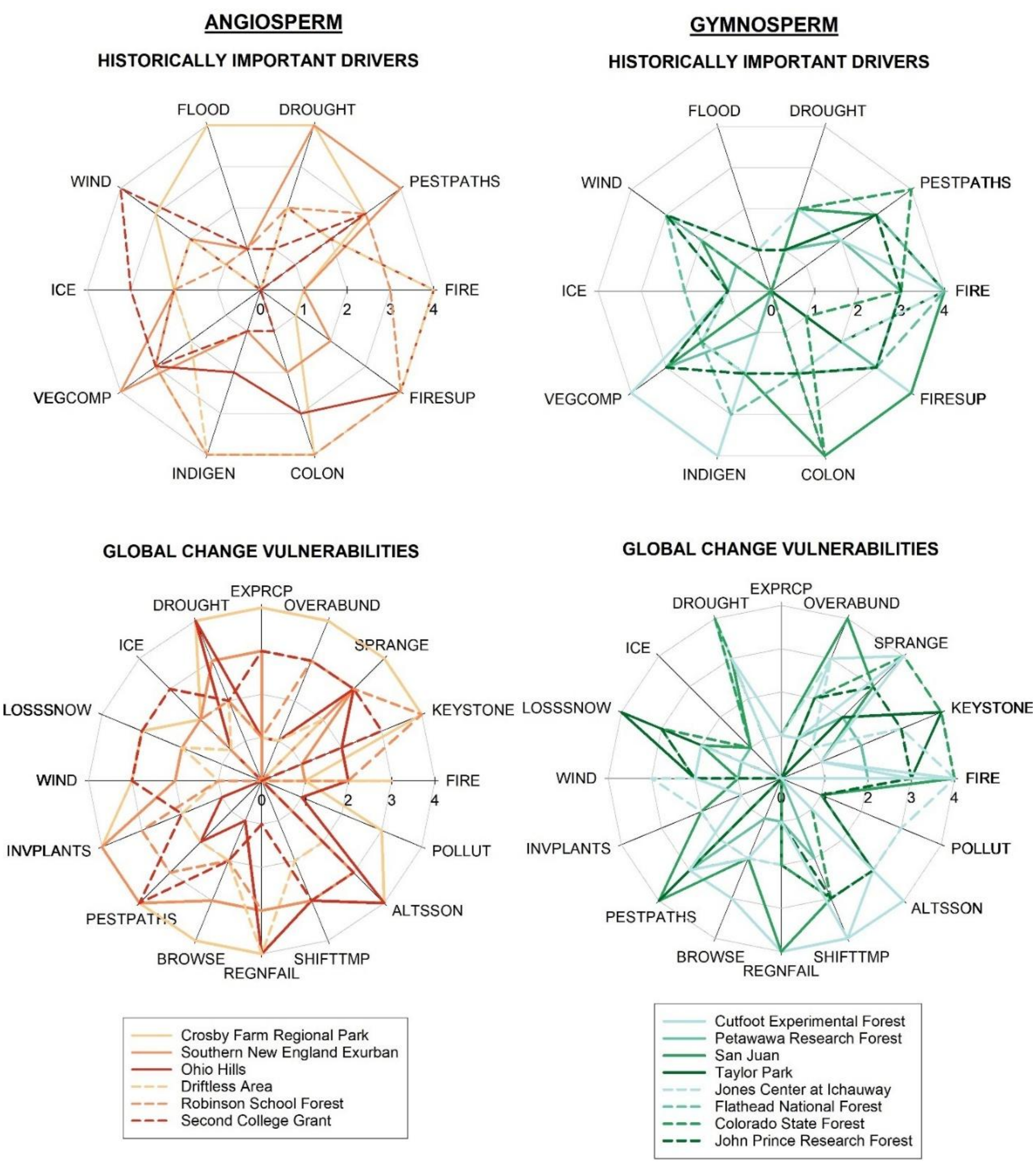

Supplement: Supplementary file 1 [file EAP-36-e70302-s001.pdf]
